# United we thrive: friendship and subsequent physical, behavioural and psychosocial health in older adults (an outcome-wide longitudinal approach)

**DOI:** 10.1017/S204579602300077X

**Published:** 2023-11-15

**Authors:** E. S. Kim, W. J. Chopik, Y. Chen, R. Wilkinson, T. J. VanderWeele

**Affiliations:** 1 Department of Psychology, University of British Columbia, Vancouver, BC, Canada; 2 Human Flourishing Program, Institute for Quantitative Social Science, Harvard University, Cambridge, MA, USA; 3 Lee Kum Sheung Center for Health and Happiness, Harvard T.H. Chan School of Public Health, Boston, MA, USA; 4 Department of Psychology, Michigan State University, East Lansing, MI, USA; 5 Department of Epidemiology, Harvard T.H. Chan School of Public Health, Boston, MA, USA; 6 Department of Biostatistics, Harvard T.H. Chan School of Public Health, Boston, MA, USA

**Keywords:** friendship, health and retirement study, outcome-wide epidemiology, physical health, psychological well-being, public health

## Abstract

**Aims:**

Three factors converge to underscore the heightened importance of evaluating the potential health/well-being effects of friendships in older adulthood. First, policymakers, scientists, and the public alike are recognizing the importance of social relationships for health/well-being and creating national policies to promote social connection. Second, many populations are rapidly aging throughout the world. Third, we currently face what some call a ‘friendship recession’. Although, growing research documents associations between friendship with better health and well-being, friendship can also have a ‘dark side’ and can potentially promote negative outcomes. To better capture friendship’s potential heterogeneous effects, we took an outcome-wide analytic approach.

**Methods:**

We analysed data from 12,998 participants in the Health and Retirement Study (HRS) – a prospective and nationally representative cohort of U.S. adults aged >50, and, evaluated if increases in friendship strength (between *t*_0_; 2006/2008 and *t*_1_; 2010/2012) were associated with better health/well-being across 35 outcomes (in *t*_2_; 2014/2016). To assess friendship strength, we leveraged all available friendship items in HRS and created a composite ‘friendship score’ that assessed the following three domains: (1) friendship network size, (2) friendship network contact frequency and (3) friendship network quality.

**Results:**

Stronger friendships were associated with better outcomes on some indicators of physical health (e.g. reduced risk of mortality), health behaviours (e.g. increased physical activity) and nearly all psychosocial indicators (e.g. higher positive affect and mastery, as well as lower negative affect and risk of depression). Friendship was also associated with increased likelihood of smoking and heavy drinking (although the latter association with heavy drinking did not reach conventional levels of statistical significance).

**Conclusions:**

Our findings indicate that stronger friendships can have a dual impact on health and well-being. While stronger friendships appear to mainly promote a range of health and well-being outcomes, stronger friendships might also promote negative outcomes. Additional research is needed, and any future friendship interventions and policies that aim to enhance outcomes should focus on how to amplify positive outcomes while mitigating harmful ones.

Three factors converge to underscore the heightened importance of evaluating the potential health and well-being effects of friendships in older adulthood. First, policymakers, scientists and the public alike are recognizing the importance of social relationships for health and well-being outcomes and creating national policies to promote social connection. For example, the United Kingdom and Japan recently appointed their first ‘ministers of loneliness’ to combat loneliness at the national level (Fried *et al.*, 2020), and the U.S. Surgeon General recently published ‘Our Epidemic of Loneliness and Isolation: The U.S. Surgeon General’s Advisory on the Healing Effects of Social Connection and Community’. Second, populations are rapidly aging in many countries throughout the world (United Nations, Department of Economic and Social Affairs, Population Division, 2020). For example, the number of people aged ≥65 years in the United States is projected to increase by nearly 50% in the next 15 years (Colby and Ortman, 2014). Coupled with this rapid pace of population aging, older adults are also more likely to experience risk factors for loneliness and social isolation, such as: living alone, loss of family and contemporaries, and illness (National Academies of Sciences, Engineering, and Medicine, 2020). Third, we are currently facing what some call a ‘friendship recession’. Even before the COVID-19 pandemic, research has shown that friendship levels have been declining over time (McPherson *et al.*, 2006; Ward, 2022). A recent report highlights our changing social landscape; in 2021, a nationwide survey found that 12% of respondents reported having 0 close friends, and 49% reported having three or fewer close friends. This contrasts markedly with 1990 when the rates were only 3% and 27%, respectively (Cox, 2021).

As populations age, identifying factors that bolster health and well-being is critical for stemming the growing wave of chronic conditions and mounting healthcare costs (Kubzansky *et al.*, 2018). Although traditional biomedical efforts have focused on identifying *risk factors* of disease (e.g. loneliness and social isolation) (Holt-Lunstad, 2022; Hong *et al.*, 2023; Valtorta *et al.*, 2016; Wilson *et al.*, 2007), researchers and policymakers increasingly seek potentially modifiable *health assets* that uniquely enhance a person’s ability to foster health and well-being (e.g. friendship) (Kubzansky *et al.*, 2018; VanderWeele, 2017). A large and growing body of research has evaluated the adverse effects of loneliness and social isolation on health and well-being outcomes. As illustrative examples, loneliness has been linked with a 50%, 29% and 26% elevated risk of developing Alzheimer’s disease, coronary heart disease and premature mortality, respectively (Holt-Lunstad, 2022; Valtorta *et al.*, 2016; Wilson *et al.*, 2007). However, much less research has focused on the potential influence that friendships exert on health and well-being outcomes, especially in older adults (Blieszner *et al.*, 2019).

Friendships are shaped by genetics, social structural factors and changing life circumstances (Blieszner *et al.*, 2019; Festinger *et al.*, 1950; Iervolino *et al.*, 2002). However, various dimensions of friendship (i.e. quantity, contact frequency, quality of interactions) can be intervened upon through several approaches (Blieszner *et al.*, 2019; Hong *et al.*, 2022) (e.g. a series of questions that foster closeness through mutual vulnerability (i.e. ‘Fast Friends’) (Aron *et al.*, 1997); an online 12-week ‘Friendship Enrichment Program’ aimed at promoting relational competence, social skills and friendship formation skills (Bouwman *et al.*, 2017); and components of other research and programs designed to reduce loneliness and social isolation) (Hoang *et al.*, 2022; Hong *et al.*, In Press). Growing research has documented friendship’s association with a range of health and well-being outcomes. Various dimensions of friendship have been associated with better: psychological health (e.g. higher life satisfaction and lower depression) (Choi *et al.*, 2020; Fowler and Christakis, 2008; Santini *et al.*, 2015), health behaviours (e.g. increased: preventive healthcare service use, physical activity, smoking cessation) (Christakis and Fowler, 2008; Han *et al.*, 2019; Watt *et al.*, 2014) and physical health (e.g. reduced risk of: cognitive decline and mortality) (Chopik, 2017; Sharifian *et al.*, 2020; Shor and Roelfs, 2015). Interestingly, like other social factors, friendship might act as an ‘amplification system’ for both beneficial and harmful health and well-being outcomes depending on the social context. Said another way, networks can magnify whatever they are seeded with (e.g. attitudes, norms, behaviours). Thus, friendships can also have a ‘dark side’ and promote negative health and well-being outcomes. However, research evaluating negative outcomes is sparse.

Existing friendship and health/well-being studies have been seminal and contributed substantially to the literature, but they remain limited from a *causal inference* perspective. First, many studies are cross-sectional, making it challenging to assess the causal and temporal sequence of variables. Second, only a small portion of studies in the field of social relationships and health have broken down results by relationship *type*, preventing us from isolating the effects of friendships. Third, among longitudinal studies evaluating friendship, many health behaviour and physical health outcomes have not been evaluated in adult populations. Fourth, some existing studies do not account for relevant potential confounders (e.g. depressive symptoms or baseline health). Fifth, existing research on friendships and health and well-being is highly fragmented. Studies often focus on a single dimension of friendship (e.g. network size, contact frequency or interaction quality) and usually limit their scope to one specific health behaviour or physical health outcome. This piecemeal approach leaves substantial gaps in our understanding of how various dimensions of friendship might collectively or differentially influence a broader range of health and well-being outcomes. Sixth, most longitudinal studies have not controlled for friendship characteristics in the pre-baseline wave. Doing so allows researchers to ask a different question – one particularly important in this era of translational research: What health and well-being outcomes might we observe within a relatively short time horizon (over 4 years) if friendships change?

To begin addressing this question we used an *outcome-wide* analytic approach (VanderWeele *et al.*, 2020). This hypothesis-generating, data-driven approach aims to discover estimates of the outcomes we might expect to observe if friendship was intervened upon. Promising findings can then undergo further investigation in future studies. We leveraged a large, prospective and nationally representative sample of U.S. older adults and tested if *changes* in friendship were associated with better subsequent health and well-being across 35 separate outcomes. These outcomes were chosen because they are frequently included in seminal gerontological models that characterize the antecedents, processes and outcomes that foster people’s ability to age well (Depp and Jeste, 2006; Rowe and Kahn, 1987; Ryff and Singer, 2009). To the best of our knowledge, this is among the first studies to evaluate how changes in friendship are associated with changes in health and well-being.

## Methods

### Study population

We used data from the Health and Retirement Study (HRS), a nationally representative panel study of adults aged >50. Beginning in 2006, HRS staff provided mail-in questionnaires to study participants and began assessing psychosocial factors in a randomly selected 50% of HRS participants. The remaining 50% of HRS participants completed the questionnaire in the subsequent wave (2008). Each sub-cohort alternates reporting so that each participant reports psychosocial data every 4 years. In 2006, the psychosocial questionnaire response rate was 88% and in 2008, it was 84% (Smith *et al.*, 2013). To increase sample size and power, we combined data from both sub-cohorts.

Our study used data from three timepoints (*t*_0_, *t*_1_, *t*_2_). All covariates (including prior levels of friendship) were assessed in the pre-baseline wave (*t*_0_; 2006/2008). The exposure – friendship – was assessed 4 years later in the baseline wave (*t*_1_; 2010/2012). All outcomes were assessed another 4 years later in the outcome wave (*t*_2_; 2014/2016). We restricted the sample to those who completed the psychosocial questionnaire at baseline because nearly half of our study outcomes were assessed in the psychosocial questionnaire, resulting in a final analytic sample size of 12,998 people.

The University of Michigan’s Institute of Social Research coordinates the study and provides extensive documentation about the protocol, instrumentation, sampling strategy and statistical weighting procedures (Smith *et al.*, 2013; Sonnega *et al.*, 2014). The HRS has been approved by several ethics committees, including the University of Michigan IRB. Further, informed consent was obtained from all HRS respondents.

### Measures

#### Friendship

We leveraged all available friendship items in HRS and created a composite ‘friendship score’ that assessed the following domains. (1) *Friendship Network Size*: was assessed by asking respondents, ‘How many of your friends would you say you have a close relationship with?’ Respondents were asked to provide a numerical answer in response. (2) *Friendship Network Contact Frequency*: was assessed by asking respondents, ‘On average, how often do you do each of the following?’ (a) ‘Meet up (include both arranged and chance meetings)’, (b) ‘Speak on the phone’, (c) ‘Write or email’. For each category, we reverse coded responses so that higher values represented more frequent contact (0 = ≤ Every Few Months, 1 = 1x-2x/Month, 2 = 1x-2x/Week, 3 = ≥ 3x/Week). (3) *Friendship Network Quality*: was assessed by asking respondents to rate perceived support and strain from their friends. On a 4-point Likert scale, respondents rated the degree to which they endorsed three support items (e.g. ‘How much can you rely on them if you have a serious problem?’) and four strain items (e.g. ‘How often do they make too many demands on you?’). Responses to all items were averaged within each dimension to create a perceived support score, and a separate strain score. To standardize the measure, we first rescored contra-indicative items and created *z*-scores for each of the items. Then, we created a composite friendship score by averaging the *z*-scores for each measure (higher scores indicated stronger friendships). To evaluate potential threshold effects, we categorized scores into quartiles based on the distribution of friendship scores in the sample. In secondary analyses, we separately evaluated each facet of the friendship composite score in relation to our health and well-being outcomes. The HRS guides (and Supplementary Text 1) provide further details about each assessment.

#### Covariates

All covariates were assessed in the pre-baseline wave (*t*_0_; 2006/2008) by self-report. Covariates included (1) demographics (age, sex, race/ethnicity [White, Black, Hispanic, Other]), marital status (married/not married), (2) annual household income (<$50,000, $50,000–$74,999, $75,000–$99,999, ≥$100,000), (3) total wealth (quintiles of the score distribution in this sample), (4) educational attainment (no degree, GED/high school diploma, ≥college degree), (5) employment (yes/no), (6) health insurance (yes/no), (7) geographic region (Northeast, Midwest, South, West), (8) religious service attendance (none, <1x/week, ≥1x/week), (9) personality (openness, conscientiousness, extraversion, agreeableness, neuroticism) and (10) childhood abuse (yes/no). We also adjusted for prior levels of friendship and all outcomes (listed directly below) in the pre-baseline wave.

#### Outcomes

We considered 35 outcomes in the outcome wave (2014/2016 (*t*_2_)), including dimensions of physical health factors (all-cause mortality, number of chronic conditions, diabetes, hypertension, stroke, cancer, lung disease, arthritis, overweight/obesity, physical functioning limitations, cognitive impairment, chronic pain and self-rated health), health behaviours (binge drinking, smoking, physical activity and sleep problems), psychological well-being (positive affect, life satisfaction, optimism, purpose in life, mastery, health mastery and financial mastery), psychological distress (depression, depressive symptoms, hopelessness, negative affect and perceived constraints) and social factors (loneliness, living with spouse/partner, frequency of contact with: children, other family or friends – each assessed separately). The HRS guides (and Supplementary Text 2) provide further details about each assessment (Fisher *et al.*, 2005; Jenkins *et al.*, 2008; Smith *et al.*, 2013; Sonnega *et al.*, 2014).

### Statistical analysis

We used an outcome-wide analytic approach (VanderWeele *et al.*, 2020), which has several characteristics not widely used outside of biostatistics and causal inference. Thus, we summarize those characteristics here.

First, we control for covariates in the wave prior to the exposure (*t*_0_), because, if we assess potential confounders in the same timepoint as the exposure (*t*_1_), it remains unclear if they are confounders or mediators. If we accidentally control for mediators in the same timepoint, we may spuriously attenuate a true effect. A pragmatic approach to avoiding this problem is to adjust for potential confounders in the pre-baseline wave (*t*_0_).

Second, to enhance our ability to strive towards “no unmeasured confounding,” and “exchangeability” (as well as other key criteria described in “disjunctive cause criterion” for selection of covariates which includes potential causes of either the exposure or the outcomes or both), which in turn enhances our ability to make causal inference, we adjust for a rich set of potential confounder variables to make these assumptions as plausible as possible (Greenland and Robins, 1986; VanderWeele *et al.*, 2020).

Third, to reduce potential reverse causality, we also adjust for all outcome variables in the pre-baseline wave (*t*_0_).

Fourth, to evaluate potential “change” in friendship, we adjust for friendship in the pre-baseline wave (*t*_0_). This helps “hold constant” pre-baseline levels of friendship (see Supplementary Text S3 for proof and further explanation). Adjusting for pre-baseline levels of friendship (*t*_0_) also has several other advantages, including reducing the risk of reverse causality by “removing” the accumulating effects that friendship already had on outcomes in the past (“prevalent exposure”) and allowing readers to instead focus on the effects of *change* in friendship (“incident exposure”), over 4 years, on outcomes.

We ran separate models for each outcome. We ran (1) logistic regression models for binary outcomes with <10% prevalence; (2) generalized linear models (with a log link and Poisson distribution) for binary outcomes with ≥10% prevalence or (3) linear regression models for continuous outcomes. We standardized all continuous outcomes (mean = 0, standard deviation = 1) so their effect size can be interpreted as a standard deviation *change* in the outcome. In our tables, and for ease of reviewing results, we present multiple *p*-value cutoffs (both with and without Bonferroni correction for multiple testing) because different investigators use different threshold standards for interpreting evidence based on current norms in their specific discipline. In our Results section, we comment on traditional 0.05 *p*-value threshold (without Bonferroni correction). However, in all cases, we also provide 95% confidence intervals which are considered preferable assessments of uncertainty since all thresholds are ultimately arbitrary.

#### Additional analyses

We ran several additional analyses. First, to evaluate the robustness of our results to potential unmeasured confounding, we conducted *E*-value analyses to assess the minimum strength of association (on the risk ratio scale) that an unmeasured confounder must have with both the exposure and the outcome to explain away the observed association (VanderWeele and Ding, 2017). Second, to evaluate how our findings might compare to past research, we re-analysed all models using a more conventional set of covariates (e.g. sociodemographics, but no control for previous friendship scores or outcomes, in the pre-baseline wave). This analytic approach asks a different question: what are the potential long-term cumulative effects that the whole history of friendship (approximated by its current measure but not controlling for the past) has on outcomes? Third, we re-analysed the main models but removed people who had any history of a given physical condition at baseline. Fourth, we separately evaluated each facet of the friendship composite score in relation to our health and well-being outcomes.

#### Multiple imputation

We imputed all missing exposures, covariates and outcomes using an imputation by chained equations approach and generated five datasets because it provides a potentially more accurate approach than other methods of handling missing data and helps address problems that arise due to attrition (Harel *et al.*, 2018). All analyses were conducted in Stata 18.0.

## Results

In the covariate wave (*t*_0_; 2006/2008), the average age of participants was 65 years old (SD = 10) and they were predominantly women (59%), married (67%) and high school educated (55%). Table 1 provides the distribution of covariates by categories of friendship. As the composite friendship score increased from the lowest quartile (Quartile 1) to the highest quartile (Quartile 4), so did the scores of its subordinate elements in a dose–response manner. For example, people in the highest versus lowest quartile reported having more close friends (7.8 vs. 1.6), meeting friends >1x/week more frequently (86.3% vs. 9.1%) and receiving more social support from friends (3.6 vs. 2.3). Supplementary Table 1 describes *change* in friendship from the pre-baseline wave (*t*_0_) to the baseline wave (*t*_1_).

**Table 1.** Characteristics of participants at baseline by quartiles of friendship (*N* = 10,087)^a^^,^^b^^,^^c^

	Friendship															
	Quartile 1 (*N* = 2,522)				Quartile 2 (*N* = 2,525)				Quartile 3 (*N* = 2,521)				Quartile 4 (*N* = 2,519)			
	Lowest quartile												Highest quartile			
Participant characteristics	*n*	%	*M*	*SD*	*n*	%	*M*	*SD*	*n*	%	*M*	*SD*	*n*	%	*M*	*SD*
**Sociodemographic factors**																
Age (yr; range: 47−97)			66.4	9.3			67.1	9.2			67.3	9.2			67.7	9.1
Female (%)	1,239	49.1			1,401	55.5			1,573	62.4			1,810	71.9		
Race/ethnicity (%)																
White	1,856	73.6			2,004	79.4			2,061	81.8			2,087	82.9		
Black	319	12.6			273	10.8			279	11.1			261	10.4		
Hispanic	273	10.8			191	7.6			128	5.1			135	5.4		
Other	74	2.9			56	2.2			53	2.1			36	1.4		
Married (%)	1,775	70.4			1,780	70.5			1,654	65.6			1,546	61.4		
Income (%)																
<$50,000	1,515	60.1			1,357	53.7			1,366	54.2			1,395	55.4		
$50,000–$74,999	425	16.9			434	17.2			418	16.6			397	15.8		
$75,000–$99,999	230	9.1			270	10.7			274	10.9			252	10.0		
≥$100,000	352	14.0			464	18.4			463	18.4			475	18.9		
Total wealth (%)																
1st Quintile	681	27.0			461	18.3			408	16.2			395	15.7		
2nd Quintile	555	22.0			506	20.0			488	19.4			468	18.6		
3rd Quintile	469	18.6			524	20.8			497	19.7			524	20.8		
4th Quintile	418	16.6			517	20.5			572	22.7			553	22.0		
5th Quintile	399	15.8			517	20.5			556	22.1			579	23.0		
Education (%)																
<High school	522	20.7			379	15.1			365	14.5			340	13.5		
High school	1,441	57.3			1,447	57.5			1,393	55.4			1,376	54.8		
≥College	553	22.0			691	27.5			756	30.1			796	31.7		
Employed (%)	1,037	41.2			1,086	43.0			1,011	40.1			979	38.9		
Health insurance (%)	2,358	93.6			2,415	95.6			2,421	96.0			2,425	96.3		
Geographic region (%)																
Northeast	359	14.2			374	14.8			366	14.5			380	15.1		
Midwest	686	27.2			758	30.0			726	28.8			627	24.9		
South	993	39.4			927	36.7			963	38.2			1,001	39.8		
West	483	19.2			464	18.4			466	18.5			509	20.2		
Childhood abuse (%)	203	8.1			154	6.2			162	6.5			165	6.6		
**Physical health**																
Diabetes (%)	523	20.7			453	17.9			413	16.4			385	15.3		
Hypertension (%)	1,398	55.4			1,349	53.4			1,351	53.6			1,307	51.9		
Stroke (%)	155	6.1			152	6.0			147	5.8			120	4.8		
Cancer (%)	318	12.6			336	13.3			349	13.8			357	14.2		
Heart disease (%)	554	22.0			517	20.5			533	21.1			447	17.7		
Lung disease (%)	244	9.7			187	7.4			183	7.3			162	6.4		
Arthritis (%)	1,429	56.7			1,471	58.3			1,472	58.4			1,447	57.4		
Overweight/obesity (%)	1,865	75.1			1,828	73.1			1,784	71.5			1,727	69.4		
Physical limitations (%)	584	23.2			479	19.0			487	19.3			383	15.2		
Cognitive impairment (%)	452	18.2			308	12.4			319	12.7			298	11.9		
Chronic pain (%)	956	37.9			834	33.0			819	32.5			757	30.1		
Self-rated health (range: 1−5)			3.1	1.1			3.3	1.0			3.4	1.0			3.5	1.0
**Health behaviours**																
Binge drinking (%)	275	13.4			289	14.3			276	13.5			231	11.3		
Smoking (%)	390	15.6			289	11.5			294	11.7			241	9.7		
Frequent physical activity (%)	1,794	71.2			1,922	76.2			2,016	80.0			1,997	79.3		
Sleep problems (%)	598	43.0			557	41.1			569	41.8			500	37.6		
Religious service attendance (%)																
Never	811	32.2			591	23.4			532	21.1			463	18.4		
<1x/week	831	33.0			841	33.3			782	31.0			765	30.4		
≥1x/week	879	34.9			1,092	43.3			1,205	47.8			1,289	51.2		
**Psychological well-being**																
Positive affect (range: 1−5)			3.4	0.8			3.6	0.7			3.7	0.7			3.9	0.7
Life satisfaction (range: 1−7)			4.7	1.5			5.1	1.4			5.2	1.4			5.5	1.4
Optimism (range: 1−6)			4.2	1.0			4.5	0.9			4.6	0.9			4.8	0.9
Purpose in life (range: 1−6)			4.4	0.9			4.6	0.9			4.8	0.9			5.0	0.8
Personal mastery (range: 1−6)			4.6	1.1			4.8	1.1			4.9	1.0			5.0	1.0
Health mastery (range: 1−10)			7.0	2.5			7.3	2.2			7.5	2.1			7.8	2.1
Financial mastery (range: 1−10)			6.9	2.8			7.3	2.5			7.6	2.4			7.8	2.4
**Psychological distress**																
Depression (%)	471	18.7			309	12.2			269	10.7			233	9.2		
Depressive symptoms (range: 0−8)			1.6	2.1			1.2	1.8			1.1	1.7			1.0	1.6
Hopelessness (range: 1−6)			2.7	1.4			2.3	1.2			2.1	1.2			1.9	1.1
Negative affect (range: 1−5)			1.8	0.7			1.7	0.6			1.6	0.6			1.5	0.5
Perceived constraints (range: 1−6)			2.5	1.2			2.1	1.1			2.0	1.1			1.8	1.0
**Social factors**																
Loneliness (range: 1−3)			1.7	0.6			1.5	0.5			1.4	0.5			1.3	0.4
Not living with spouse/partner (%)	632	25.7			643	26.1			757	30.6			880	35.9		
Contact children ≥1x/week (%)	1,707	69.3			1,857	75.2			1,903	76.9			1,950	79.6		
Contact other family ≥1x/week (%)	1,055	42.5			1,277	51.1			1,336	53.5			1,519	61.3		
**Personality**																
Openness (range: 1−4)			2.8	0.5			2.9	0.5			3.0	0.5			3.1	0.5
Conscientiousness (range: 1−4)			3.3	0.5			3.4	0.4			3.4	0.4			3.5	0.4
Extraversion (range: 1−4)			3.0	0.6			3.1	0.5			3.3	0.5			3.4	0.5
Agreeableness (range: 1−4)			3.4	0.5			3.5	0.5			3.6	0.4			3.7	0.4
Neuroticism (range: 1−4)			2.2	0.6			2.1	0.6			2.0	0.6			1.9	0.6
**Friendship factors**																
# of close friends (range: 0−124)			1.6	2.0			3.2	2.6			4.3	3.6			7.8	9.4
Meetup with friends ≥1x/week (%)	176	9.1			782	31.1			1447	57.9			2047	83.6		
Call friends ≥1x/week (%)	228	11.7			1012	40.3			1851	74.1			2310	94.3		
Writing friends ≥1x/week (%)	33	1.8			191	8.0			471	19.8			1134	53.0		
Friend social support (range: 1−4)			2.3	0.6			2.9	0.6			3.3	0.6			3.6	0.5
Friend social strain (range: 1−4)			1.6	0.6			1.5	0.5			1.4	0.4			1.3	0.4

a This table was created based on non-imputed data.

b All of these variables were used as covariates, and assessed in the wave prior (2006/2008) to the exposure wave (2010/2012).

c The percentages in some sections may not add up to 100% due to rounding.

Table 2 shows the associations between friendship and subsequent health and well-being outcomes over the 4-year follow-up period. When considering physical health outcomes, friendship was associated with 3 (out of 14) outcomes, including 24% reduced risk of all-cause mortality (95% confidence interval [CI]: 0.62, 0.95), 19% reduced risk of stroke (95% CI: 0.67, 0.97), and higher self-rated health (*β* = 0.09, 95% CI: 0.04, 0.15). When considering health behaviours, friendship was associated with 2 (out of 4) outcomes including a 43% (95% CI: 1.03, 1.99) increased likelihood of smoking and 9% increased likelihood of frequency physical activity (95% CI: 1.00, 1.18).

**Table 2.** Friendship and subsequent health and well-being (health and retirement study [HRS]: *N* = 12,998)^a^^,^^b^^,^^c^^,^^d^

	Friendship			
	Quartile 1 (*n* = 3,249)	Quartile 2 (*n* = 3,246)	Quartile 3 (*n* = 3,246)	Quartile 4 (*n* = 3,247)
Outcomes	(Reference)	RR/OR/*β* (95% CI)	RR/OR/*β* (95% CI)	RR/OR/*β* (95% CI)
**Physical Health**				
All-cause mortality	1.00	0.96 (0.81, 1.14)	0.85 (0.71, 1.01)	0.76 (0.62, 0.95)*
Number of chronic conditions	0.00	−0.01 (−0.04, 0.02)	−0.02 (−0.05, 0.02)	−0.01 (−0.05, 0.02)
Diabetes	1.00	0.98 (0.89, 1.09)	1.01 (0.92, 1.12)	0.96 (0.86, 1.09)
Hypertension	1.00	1.01 (0.95, 1.07)	1.01 (0.94, 1.08)	1.01 (0.94, 1.09)
Stroke	1.00	0.94 (0.81, 1.10)	0.86 (0.73, 1.01)	0.81 (0.67, 0.97)*
Cancer	1.00	1.01 (0.90, 1.13)	1.04 (0.92, 1.18)	1.08 (0.94, 1.23)
Heart disease	1.00	0.99 (0.90, 1.08)	1.01 (0.91, 1.11)	0.99 (0.89, 1.10)
Lung disease	1.00	0.98 (0.85, 1.12)	0.98 (0.84, 1.15)	0.95 (0.79, 1.14)
Arthritis	1.00	1.00 (0.94, 1.06)	1.00 (0.94, 1.07)	1.00 (0.93, 1.08)
Overweight/obesity	1.00	1.00 (0.94, 1.06)	0.98 (0.92, 1.04)	0.99 (0.92, 1.07)
Physical functioning limitations	1.00	0.95 (0.86, 1.05)	0.95 (0.86, 1.05)	0.90 (0.80, 1.00)
Cognitive impairment	1.00	0.93 (0.84, 1.03)	0.90 (0.80, 1.01)	0.91 (0.79, 1.04)
Chronic pain	1.00	0.99 (0.90, 1.08)	1.00 (0.91, 1.10)	1.01 (0.90, 1.13)
Self-rated health	0.00	0.04 (−0.01, 0.09)	0.05 (0.00, 0.10)*	0.09 (0.04, 0.15)**
**Health Behaviours**				
Heavy drinking	1.00	1.22 (0.98, 1.52)	1.31 (0.91, 1.90)	1.48 (0.96, 2.30)
Smoking	1.00	1.11 (0.80, 1.54)	1.32 (0.97, 1.80)	1.43 (1.03, 1.99)*
Frequent physical activity	1.00	1.03 (0.96, 1.10)	1.06 (0.99, 1.13)	1.09 (1.00, 1.18)*
Sleep problems	1.00	0.98 (0.90, 1.08)	1.02 (0.92, 1.12)	1.05 (0.94, 1.17)
**Psychological Well-Being**				
Positive affect	0.00	0.08 (0.04, 0.12)**	0.13 (0.08, 0.19)**	0.22 (0.15, 0.28)**
Life satisfaction	0.00	0.05 (−0.00, 0.10)	0.09 (0.04, 0.14)**	0.17 (0.10, 0.23)**
Optimism	0.00	0.08 (0.01, 0.14)*	0.11 (0.03, 0.19)*	0.18 (0.09, 0.27)**
Purpose in life	0.00	0.08 (0.02, 0.13)**	0.12 (0.07, 0.18)**	0.17 (0.12, 0.23)**
Mastery	0.00	0.11 (0.04, 0.19)**	0.14 (0.08, 0.20)**	0.20 (0.14, 0.27)**
Health mastery	0.00	0.05 (0.01, 0.10)*	0.08 (0.03, 0.13)**	0.11 (0.03, 0.19)*
Financial mastery	0.00	0.09 (0.02, 0.17)*	0.13 (0.06, 0.21)**	0.15 (0.06, 0.23)**
**Psychological Distress**				
Depression	1.00	0.90 (0.76, 1.07)	0.92 (0.78, 1.09)	0.83 (0.68, 1.00)*
Depressive symptoms	0.00	−0.07 (−0.12, − 0.02)**	−0.05 (−0.10, − 0.00)*	−0.09 (−0.15, − 0.04)**
Hopelessness	0.00	−0.09 (−0.16, − 0.03)**	−0.10 (−0.17, − 0.03)**	−0.14 (−0.21, − 0.07)**
Negative affect	0.00	−0.11 (−0.16, − 0.07)**	−0.15 (−0.20, − 0.10)**	−0.20 (−0.26, − 0.13)**
Perceived constraints	0.00	−0.09 (−0.14, − 0.04)**	−0.09 (−0.15, − 0.04)**	−0.15 (−0.21, − 0.09)**
**Social Factors**				
Loneliness	0.00	−0.09 (−0.15, − 0.02)*	−0.13 (−0.19, − 0.08)**	−0.21 (−0.29, − 0.13)**
Not living with a spouse/partner	1.00	1.08 (0.99, 1.17)	1.11 (1.02, 1.21)*	1.20 (1.09, 1.32)**
Contact children ≥ 1x/week	1.00	1.03 (0.96, 1.09)	1.02 (0.95, 1.09)	1.02 (0.95, 1.10)
Contact other family ≥ 1x/week	1.00	1.11 (1.01, 1.22)*	1.15 (1.05, 1.25)**	1.18 (1.07, 1.31)**
Contact friends ≥ 1x/week	1.00	1.47 (1.35, 1.61)**	1.78 (1.63, 1.94)**	1.96 (1.77, 2.17)**

Abbreviations: CI, confidence interval; OR, odds ratio; RR, risk ratio.

a If the reference value is “1,” the effect estimate is OR or RR; if the reference value is “0,” the effect estimate is *β*.

b The analytic sample was restricted to those who had participated in the baseline wave (*t*_1_; 2010 or 2012). Multiple imputation was performed to impute missing data on the exposure, covariates and outcomes. All models controlled for sociodemographic characteristics (age, sex, race/ethnicity, marital status, annual household income, total wealth, level of education, employment status, health insurance, geographic region), pre-baseline childhood abuse, pre-baseline religious service attendance, pre-baseline values of the outcome variables (diabetes, hypertension, stroke, cancer, heart disease, lung disease, arthritis, overweight/obesity, physical functioning limitations, cognitive impairment, chronic pain, self-rated health, binge drinking, current smoking status, physical activity, sleep problems, positive affect, life satisfaction, optimism, purpose in life, mastery, health mastery, financial mastery, depressive symptoms, hopelessness, negative affect, perceived constraints, loneliness, living with spouse/partner, contact children <1x/week, contact other family <1x/week, contact friends <1x/week), personality factors (openness, conscientiousness, extraversion, agreeableness, neuroticism) and the pre-baseline value of the exposure. These variables were controlled for in the pre-baseline was (in t0; 2006 or 2008).

c We used an outcome-wide analytic approach and ran a separate model for each outcome. We ran a different type of model depending on the nature of the outcome: (1) for each binary outcome with a prevalence of ≥10%, we used a generalized linear model (with a log link and Poisson distribution) to estimate a RR; (2) for each binary outcome with a prevalence of <10%, we used a logistic regression model to estimate an OR; and (3) for each continuous outcome, we used a linear regression model to estimate a *β*.

d All continuous outcomes were standardized (mean = 0; standard deviation = 1), and *β* was the standardized effect size.

* p < 0.05 before Bonferroni correction; **p < 0.01 before Bonferroni correction; ***p < 0.05 after Bonferroni correction (the *p*-value cutoff for Bonferroni correction is *p* = 0.05/35 outcomes = p < 0.001).

Friendship was associated with all 7 (out of 7) psychological well-being outcomes. The strongest associations were with higher: positive affect (*β* = −0.22, 95% CI: 0.15, 0.28) and sense of mastery (*β* = 0.20, 95% CI: 0.14, 0.27). Friendship was also associated with all 5 (out of 5) psychological distress outcomes. The strongest associations were with negative affect (*β* = −0.20, 95% CI: −0.26, −0.13) and a 17% reduced risk of depression (95% CI: 0.68, 1.00). Finally, friendship was associated with all 5 (out of 5) social factors. The strongest associations were with 96% increased likelihood of contacting friends ≥1x/week (95% CI: 1.77, 2.17) and 20% increased likelihood of not living with a spouse/partner (95% CI: 1.09, 1.32).

### Additional analyses

We conducted several additional analyses. First, *E*-value analyses suggested that a few of the associations we observed were at least moderately robust to unmeasured confounding (Table 3). For example, an unmeasured confounder of both friendship and all-cause mortality by risk ratios of 1.94, each, above and beyond the large number of potential confounders already adjusted for, could explain away the association. However, weaker joint confounder associations could not. To shift the confidence interval to include the null, an unmeasured confounder associated with both friendship and all-cause mortality by risk ratios of 1.30 each could suffice, but weaker joint confounder associations could not. Several other associations were not especially robust to potential unmeasured confounding. Second, conventionally adjusted covariate models generally showed larger coefficients than fully-adjusted models (Supplementary Table 2). These analytic differences might emphasize the effect of short-term *change* in friendship vs. accumulating effects over time or might reflect residual confounding in conventional analyses. Third, when re-evaluating the fully-adjusted models after removing anyone with a history of a given physical condition at baseline (*t*_1_), the coefficients were generally larger (top panel of Supplementary Table 2). When evaluating each dimension of the friendship composite score, frequency of meeting with friends and negative social strain from friends appeared to have the largest coefficients (Supplementary Tables 3–8).

**Table 3.** Robustness to unmeasured confounding (*E*-values) for the associations between friendship (4th quartile vs. 1st quartile) and subsequent health and well-being (*N* = 12,998)^a^

	Effect estimate^b^	Confidence interval limit^c^
**Physical Health**		
All-cause mortality	1.94	1.30
Number of chronic conditions	1.13	1.00
Diabetes	1.23	1.00
Hypertension	1.10	1.00
Stroke	1.77	1.20
Cancer	1.36	1.00
Heart disease	1.11	1.00
Lung disease	1.29	1.00
Arthritis	1.07	1.00
Overweight/obesity	1.09	1.00
Physical functioning limitations	1.47	1.00
Cognitive impairment	1.44	1.00
Chronic pain	1.11	1.00
Self-rated health	1.39	1.22
**Health Behaviours**		
Binge drinking	2.33	1.00
Smoking	2.22	1.22
Frequent physical activity	1.40	1.07
Sleep problems	1.28	1.00
**Psychological Well-being**		
Positive affect	1.74	1.58
Life satisfaction	1.60	1.44
Optimism	1.63	1.42
Purpose in life	1.62	1.47
Mastery	1.69	1.52
Health mastery	1.44	1.22
Financial mastery	1.55	1.33
**Psychological Distress**		
Depression	1.72	1.06
Depressive symptoms	1.39	1.22
Hopelessness	1.52	1.34
Negative affect	1.68	1.52
Perceived constraints	1.55	1.40
**Social Factors**		
Loneliness	1.72	1.53
Living with spouse/partner	1.69	1.40
Contact children ≥1x/week	1.17	1.00
Contact other family ≥1x/week	1.65	1.35
Contact friends ≥1x/week	3.32	2.93

a See VanderWeele and Ding (2017) for the formula for calculating *E*-values.

b The *E*-values for effect estimates are the minimum strength of association on the risk ratio scale that an unmeasured confounder would need to have with both the exposure and the outcome to fully explain away the observed association between the exposure and outcome, conditional on the measured covariates.

c The *E*-values for the limit of the 95% confidence interval (CI) closest to the null denote the minimum strength of association on the risk ratio scale that an unmeasured confounder would need to have with both the exposure and the outcome to shift the confidence interval to include the null value, conditional on the measured covariates.

## Discussion

In a large, diverse, prospective and nationally representative sample of people aged >50, we observed that stronger friendships were associated with some indicators of better: physical health outcomes (i.e. reduced risk of: stroke and mortality) and health behaviours (i.e. increased physical activity), as well as better outcomes on psychological well-being (i.e. increased: positive affect, life satisfaction, optimism, purpose in life, mastery, health mastery, financial mastery), psychological distress (i.e. decreased: depression, depressive symptoms, hopelessness, negative affect, perceived constraints) and social factors (e.g. better scores on: loneliness, living with spouse, contact with other family and friends). Friendship was not associated with other physical health outcomes and health behaviours. It was also associated with *increased* likelihood of smoking and heavy drinking (although the latter association did not reach conventional levels of statistical significance).

Our results both align with and deviate from results from past work that evaluated associations between the “prevalence” of friendship and outcomes. For example, consistent with past research we observed that “incident” friendship was associated with some better outcomes: psychological well-being (e.g. increased: life satisfaction and lower depression) (Choi *et al.*, 2020; Fowler and Christakis, 2008; Santini *et al.*, 2015), health behaviours (e.g. increased: physical activity and heavy drinking) (Rosenquist *et al.*, 2010; Watt *et al.*, 2014) and physical health (e.g. reduced risk of mortality) (Shor and Roelfs, 2015). However, our results also diverge with results from past research. For example, we did not observe associations with some physical health outcomes (e.g. reduced risk of cognitive impairment, lower number of physical health conditions) (Chopik, 2017; Sharifian *et al.*, 2020) that past studies observed. However, when considering our results that adjusted for conventional covariates, we observed associations that align with this prior research.

Methodologically, the underlying reasons for diverging results may stem from a range of sources including differences in: (1) which covariates were controlled for, (2) control for prior friendship scores, (3) study population (e.g. nationally representative vs. non-generalizable samples, younger vs. older samples), (4) study design (e.g. cross-sectional vs. longitudinal), (5) measurement of the exposure (friendship has been measured in different ways), (6) measurement of the outcome (e.g. specific outcome vs. composite measures) and (7) type of analyses employed (e.g. sociometric analyses vs. covariate-controlled regression approach). When evaluating results from conventionally adjusted models, many associations observed in the friendship literature were also observed in our results. This suggests that our analyses emphasize short-term change in friendship vs. accumulating effects over longer durations of time.

We also observed that friendship was associated with a few adverse outcomes. For example, it was associated with a 43% increased likelihood of smoking and 48% increased likelihood of heavy drinking (this latter association did not reach conventional levels of statistical significance but does align with results from some prior research) (Rosenquist *et al.*, 2010). Like other social factors, friendship might act as an “amplification system” for both beneficial and harmful health and well-being outcomes depending on the social context by magnifying whatever they are seeded with (e.g. attitudes, norms, behaviours). Thus, friendships can also have a “dark side” and promote negative health and well-being outcomes by (Christakis and Fowler, 2007; Villalonga-Olives and Kawachi, 2017): (1) excessively straining group members by “requiring” them to provide support to others, (2) restricting freedom because of excessive informal control, (3) excluding out-group members, (4) “down-levelling” norms so that individuals trying to break free from negative group norms are penalized and (5) facilitating the “contagion” of unhealthy behaviours from negative role models. As an illustration of friendship’s dualistic nature on health outcomes, friends can be a source of emotional and social support for one another, yet if the exchange of support occurs in social contexts where there is smoking and/or excessive consumption of alcohol, the impact of friendship on health and well-being outcomes may be both positive and negative.

However, the majority of our results suggest that friendship has a salubrious association with health and well-being outcomes. Several hypothesized mechanisms illustrate how friendships might promote health and well-being (Blieszner *et al.*, 2019; Holt-Lunstad, 2022; Hong *et al.*, 2022; Kawachi *et al.*, 2008; Villalonga-Olives and Kawachi, 2017). Friendships might promote health by: (1) increased diffusion of information about health (e.g. referrals to high-quality healthcare practitioners); (2) social and psychological support (e.g. instrumental and emotional support in times of distress); (3) maintenance of healthy norms through informal social control (e.g. reinforcing norms that certain behaviours [e.g. exercising] are desirable and the norm) and (4) providing companionship, fun and satisfaction through mutual interests and shared activities.

Our study has several limitations, including potential self-report and common method bias, as both friendship and nearly all outcomes were self-reported. However, control for pre-baseline outcomes and a wide range of potential confounders helps to mitigate these concerns. Confounding by unmeasured variables and reverse causality are common concerns in observational research. However, controlling for a large array of variables, including the exposure in the pre-baseline wave, the prospective nature of our data, and results from *E*-value analyses helps mitigate these concerns. Our results could have been influenced (moderated) by numerous other factors such as: marital status, age, sex, race/ethnicity, socioeconomic status, personality, baseline health and others. Future work should formally evaluate these potential moderators of the friendship and health/well-being association. Our study also featured several strengths, including the use of a large, diverse, prospective and nationally representative sample of older adults. Further, we attained stronger evidence of causality for our question of interest because we adjusted for pre-baseline values of the exposure and outcomes, as well as a robust range of covariates (VanderWeele *et al.*, 2020).

Policymakers, scientists and the public alike are recognizing the importance of social relationships for health and well-being outcomes, and many countries have even begun creating national policies to strengthen social bonds. Findings from our study suggest that friendships are an important element to consider in these efforts. Further, continuously iterating existing interventions that target friendship (Aron *et al.*, 1997; Blieszner *et al.*, 2019; Bouwman *et al.*, 2017; Hoang *et al.*, 2022; Hong *et al.*, 2022), and creating new interventions might be a promising method of enhancing several aspects of health and well-being in our rapidly aging population.

## Supporting information

Kim et al. supplementary material Kim et al. supplementary material

## Data Availability

Data are available for download at https://hrsdata.isr.umich.edu/data-products.

## Supplementary material

The supplementary material for this article can be found at https://doi.org/10.1017/S204579602300077X.
