# Supplementary material for: United we thrive: friendship and subsequent physical, behavioural and psychosocial health in older adults (an outcome-wide longitudinal approach)
Source: Epidemiol Psychiatr Sci. 2023 Nov 15;32:e65. doi: 10.1017/S204579602300077X (PMC10689060; doi:10.1017/S204579602300077X)
Supplement: Kim et al. supplementary material [file S204579602300077Xsup001.docx]

**Supplementary Material**

**Appendix Text 1.** Assessment of Friendship

**Appendix Text 2.** Assessment of Outcomes

**Appendix Text 3.** Proof Illustrating How Adjusting for Pre-Baseline Levels of Friendship Can Help Us Evaluate How “Change” in Friendship is Associated with Subsequent Health and Well-Being Outcomes Over Time

**Appendix Table 1.** Change in Friendship from the Pre-Baseline Wave (t_0_) to the Baseline Wave (t_1_)

**Appendix Table 2.** Friendship and Subsequent Health/Well-Being (Adjustment for Conventional/All Covariates: N=12,998)

**Appendix Table 3.** Friendship Network Size and Subsequent Health/Well-Being (Health and Retirement Study [HRS]: N=12,998)

**Appendix Table 4.** Friendship Network Contact Frequency (In-Person Meetups) and Subsequent Health/Well-Being (Health and Retirement Study [HRS]: N=12,998)

**Appendix Table 5.** Friendship Network Contact Frequency (Calls) and Subsequent Health/Well-Being (Health and Retirement Study [HRS]: N=12,998)

**Appendix Table 6.** Friendship Network Contact Frequency (Writing) and Subsequent Health/Well-Being (Health and Retirement Study [HRS]: N=12,998)

**Appendix Table 7.** Friendship Network Quality (Positive Social Support from Friends) and Subsequent Health/Well-Being (Health and Retirement Study [HRS]: N=12,998)

**Appendix Table 8.** Friendship Network Quality (Negative Social Strain from Friends) and Subsequent Health/Well-Being (Health and Retirement Study [HRS]: N=12,998)

**Appendix Text 1**

**Assessment of Friendship**

*Friendship Network Size.* Friendship network size was assessed by asking respondents, “How many of your friends would you say you have a close relationship with?” Respondents were asked to provide a numerical answer in response.

*Friendship Network Contact Frequency*. Friendship network contact frequency was assessed by asking respondents, “On average, how often do you do each of the following?” a) “Meet up (include both arranged and chance meetings),” b) “Speak on the phone,” c) “Write or email.”

For each category, we reverse coded responses so that higher values represented more frequent contact (0=≤Every Few Months, 1=1x-2x/Month, 2=1x-2x/Week, 3=≥3x/Week).

*Friendship Network Quality*. Friendship network quality was assessed by asking respondents to rate perceived support and strain from their friends. On a 4-point Likert scale (from 1 (A lot) to 4 (Not at all), respondents rated the degree to which they endorsed three support items (e.g., “How much can you rely on them if you have a serious problem?,” “How much do they really understand the way you feel about things?,” “How much can you open up to them if you need to talk about your worries?”) and four strain items (e.g., “How often do they make too many demands on you?,” “How much do they criticize you?,” “How much do they let you down when you are counting on them?,” “How much do they get on your nerves?”). Responses to all items were averaged within each dimension to create a perceived support score (α=0.84, range 1-4), and a separate strain score (α=0.76, range 1-4).

To standardize the measure, we first rescored contra-indicative items and created z-scores for each of the items. Then, we created a composite friendship score by averaging the z-scores for each measure (higher scores indicated stronger friendships). To evaluate potential threshold effects, we categorized scores into quartiles based on the distribution of friendship scores in the sample. In secondary analyses, we separately evaluated each facet of the friendship composite score in relation to our health and well-being outcomes.

**Appendix Text 2**

**Assessment of Outcomes**

**Reference Group**

The reference group was the healthiest group for all binary outcomes unless otherwise noted.

**Physical Health**

*All-cause mortality*. Two methods were used to obtain information about death up to the 2016 questionnaire wave: 1) An exit interview was conducted with next-of-kin. 2) The National Death Index (NDI) was searched for death information after each wave of data collection. There is a 95.5% match when comparing deaths reported by exit interviews versus the NDI.^1^

*Chronic conditions.* Participants self-reported (yes/no) if they were ever told by a healthcare provider that they had the following conditions: 1) diabetes, 2) hypertension, 3) stroke, 4) cancer, 5) heart disease, 6) lung disease, or 7) arthritis. The HRS has demonstrated validity and reliability of self-reported chronic conditions.^2^

*Overweight/obesity*. Body mass index (BMI) was derived from self-reported height and weight. It was calculated as weight/height^2^ (kg/m^2^). A BMI of ≥25 kg/m^2^ was considered as overweight/obese.^3^

*Number of chronic conditions.* To create a score for the number of chronic conditions, a summary score was calculated by summing the number of reported conditions. This measure included the 7 chronic conditions above and also overweight/obesity (range 0-8).

*Cognitive functioning limitations*. The HRS cognitive functioning assessment^4,5^ was adapted from the modified Telephone Interview for Cognitive Status (TICS-M). The assessment included an immediate and delayed 10-noun free recall test, a serial 7 subtraction test, and a backward count 20 test (27-point scale overall). This assessment tool has been shown to have high sensitivity and specificity when assessing cognitive impairment in older adults. The cutpoints used in this study were derived from previous research on cognitive impairment in HRS.^6,7^ Participants who scored 0-11 (on the 27-point scale) were classified as having “cognitive impairment”, while participants scoring ≥12 were classified as “normal” (the reference group). HRS reports contain further information about these cognitive assessments.^4,5^

*Physical functioning limitations.* Physical functioning limitations were assessed using items from scales developed by Rosow and Breslau (1966), Nagi (1976), Katz, Ford, Moskowitz, Jackson, and Jaffe (1963), and Lawton and Brody (1969). A total of 15 questions about physical functioning (e.g., walking several blocks, climbing one flight of stairs, pushing or pulling large objects, lifting or carrying 10 pounds, getting up from a chair, reaching or extending arms up, stooping, kneeling, or crouching, sitting for 2 hours) and activities of daily living (e.g., walking across a room, dressing, eating, bathing, getting in/out bed, using the toilet, picking up a dime) were included. Participants were classified as having “physical functioning limitations” if they reported >4 limitations with physical functioning, while participants who reported <4 limitations were considered “normal” (the reference group). This criterion was determined by identifying the physical function score where 75% of participants could be considered as having healthy physical function at baseline.

*Chronic pain*. Participants were asked (yes/no): “Are you often troubled with pain?” The reference group was no pain.

*Self-rated health.* Participants were asked, “Would you say your health is excellent, very good, good, fair, or poor?” on a 5-point scale (reverse coded with higher scores indicating higher self-rated health).

**Health Behaviors**

*Heavy drinking.* Following the National Institute on Alcohol Abuse and Alcoholism guidelines,^8^ heavy drinking was defined as >14 for drinks/week for men and >7 drinks/week for women. Alcohol consumption was measured by multiplying the number of days/week that alcohol was consumed x number of drinks/day, which resulted in the number of drinks/week. Participants not in this alcohol consumption range were classified as non-heavy drinkers (the reference group).

*Smoking*. Participants were asked (yes/no): “Do you smoke cigarettes now?” to assess current smoking status. The reference group was “no” smoking.

*Frequent physical activity*. Based on prior research, a binary physical activity variable was created: ≥1x/week of vigorous or moderate exercise was considered frequent physical activity, while <1x/week of vigorous or moderate exercise was the reference group.^9^ Participants indicated the frequency (i.e., response categories: daily, >1x/week, 1x/week, 1-3x/month, hardly ever or never) with which they engaged in vigorous (e.g., running, swimming, aerobics), moderate (e.g., gardening, dancing, walking at a moderate pace), and light (e.g., vacuuming, laundry) activities over the past 12 months.

*Sleep problems.* Participants completed the 4-item Jenkins Sleep Questionnaire, a widely used and validated screening instrument for assessing sleep complaints and insomnia symptoms.^10^ Response categories included “most of the time,” “sometimes,” and “rarely or never.” Healthy sleep (no sleep problems) was defined as reporting “rarely or never” for all four insomnia symptoms assessed (the reference group). People who responded “most of the time” to any of the items were categorized as having sleep problems, and the final results were reverse coded. The sleep questionnaire was only administered every other wave. Thus, sleep data was imputed for half of the sample. Imputed and complete-case analyses showed similar estimates.

**Psychological Well-Being**

*Positive affect*. Positive affect was assessed with a 13-item measure based on the Positive and Negative Affect Schedule (PANAS-X).^11^ It included the following items: determined, enthusiastic, active, proud, interested, happy, attentive, content, inspired, hopeful, alert, calm, excited, with response categories ranging from 1 (all of the time) to 5 (none of the time). Responses to these 13 items were averaged to obtain an overall score (α=0.92, range 1-5).

*Life satisfaction.* Life satisfaction was assessed with the 5-item Satisfaction with Life Scale.^12^ The scale has shown excellent psychometric properties in prior work. Using a 7-point Likert scale (from 1 (strongly disagree) to 7 (strongly agree)), participants were asked the extent to which they agreed with statements such as, “In most ways my life is close to ideal.” Responses to all items were averaged to create a composite score, with a higher score indicating higher life satisfaction (α=0.88, range 1-7).

*Optimism.* Optimism was assessed with the Life Orientation Test-Revised (LOT-R), which has good discriminant and convergent validity, as well as good reliability.^13^ Using a 6-point Likert scale (from 1 (strongly disagree) to 6 (strongly agree)), participants were asked the degree to which they agreed with statements such as, “In uncertain times, I usually expect the best.” Negatively worded items were reverse coded and responses to all items were averaged to create an overall score, with a higher score indicating higher optimism (α=0.75, range 1-6).

*Purpose in life.* Purpose in life was assessed with a 7-item purpose in life subscale from the Ryff’s Psychological Well-Being Scale.^14^ The 7-item subscale has been validated in prior work and has shown good psychometric properties.^15^ Using a 6-point Likert scale (from 1 (strongly disagree) to 6 (strongly agree)), participants were asked the degree to which they agreed with statements such as, “I have a sense of direction and purpose in my life.” Negatively worded items were reverse coded and all items were averaged to create a composite score, with a higher score indicating higher purpose (α=0.75, range 1-6).

*Mastery.* Mastery was assessed with 5-items derived from Lachman and Weaver (1998). The measure has good discriminant and convergent validity, and good reliability. Using a 6-point Likert scale (from 1 (strongly disagree) to 6 (strongly agree)), participants were asked the degree to which they agreed with statements such as, “I can do just about anything I really set my mind to.” All items were averaged to create a composite score, with higher scores indicating higher mastery (α=0.90, range 1-6).

*Perceived constraints.* Perceived constraints were assessed with 5 other items derived from Lachman and Weaver (1998), and this measure has good discriminant and convergent validity, as well as good reliability. Using a 6-point Likert scale (from 1 (strongly disagree) to 6 (strongly agree)), participants were asked the degree to which they agreed with statements such as, “What happens in my life is often beyond my control.” All items were averaged to create an overall score, with higher scores indicating a higher sense of constraints on personal control (α=0.86, range 1-6).

*Health mastery.* Participants were asked, “How would you rate the amount of control you have over your health these days?” on a 0 (“no control at all”) to 10 (“very much control”) scale.

*Financial mastery.* Participants were asked, “How would you rate the amount of control you have over your financial situation these days?” on a 0 (“no control at all”) to 10 (“very much control”) scale.

**Psychological Distress**

*Depressive symptoms and depression.* Depressive symptoms were measured using The Center for Epidemiologic Studies Depression Scale (CESD).^16^ Participants indicated the presence of 8 depressive symptoms (e.g., “Much of the time during the past week, I felt depressed”) over the past week (yes/no). All items were summed, with a higher score indicating higher depressive symptoms (α=0.80, range 0-8). This scale has been validated in the HRS.^17^ Participants with a score of ≥4 were classified as having depression, as done previously (no depression was the reference group).^17^ Prior work has suggested that the cutoff value of 4 would produce results similar to the 16-item cutoff when using the full (20-item) CESD scale.^17^

*Hopelessness.* Hopelessness was assessed with a 4-item questionnaire from two previously validated scales.^18,19^ Using a 6-point Likert scale (from 1 (strongly disagree) to 6 (strongly agree)), participants were asked the degree to which they agree with statements such as, “The future seems hopeless to me and I can’t believe that things are changing for the better.” All items were averaged to create a composite score (α=0.86, range 1-6).

*Negative affect*. Negative affect was assessed with a 12-item measure based on the Positive and Negative Affect Schedule (PANAS-X).^11^ It included the following items: afraid, upset, guilty, scared, frustrated, bored, hostile, jittery, ashamed, nervous, sad, distressed, with response categories ranging from 1 (all of the time) to 5 (none of the time). Responses to these 12 items were averaged to obtain an overall score (α=0.89, range 1-5).

**Social Factors**

*Loneliness.* Loneliness was assessed with three items from the previously validated UCLA Loneliness Scale.^20^ Participants answered the following questions: How much of the time do you feel: 1) you lack companionship, 2) left out, and 3) isolated from other, with response categories ranging from 1 (often) to 3 (hardly ever or never). Responses were reverse scored and averaged, with a higher score indicated higher loneliness (α=0.80, range 1-3).

*Living with partner/spouse.* Participants were asked, “Do you have a husband, wife, or partner with whom you live?”, and answered yes/no.

*Frequency of Contact with: Children, Other Family, and Friends.* Frequency of contact was measured as the frequency with which participants were in contact with their children, other family, or friends (separately). Participants were asked, “On average, how often do you do each of the following?” 1) “Meet up (include both arranged and chance meetings),” 2) “Speak on the phone,” 3) “Write or email,” and had the choice of the following 6 responses: 1) ≥3x/week, 2) 1x-2x/week, 3) 1-2x/month, 4) every few months, 5) 1-2x/year, 6) <1x/year or never.^21^ The highest value on any of the three modes of contact was taken for each relationship type, since contact (regardless of the mode of contact) was the main point of interest. For example, if the respondent did not speak on the phone very often with a given person but met them in person very often, contact was operationalized as being common. Two categories of contact were created: 1) frequent contact: ≥1x/week contact (the reference group) and 2) infrequent contact: <1x/week of contact.

**Other Factors**

*Personality*. Personality was assessed with 26 items derived from the Midlife Development Inventory Personality scales (MIDI) and International Personality Item Pool (IPIP): the “Big-5” personality traits (openness to experience, conscientiousness, extraversion, agreeableness, and neuroticism),^22^ The goal of MIDI was to create the shortest possible measure that assessed the Big-5 personality traits with high validity and reliability using existing trait inventories. In a pilot study with a probability sample of 1,000 adults aged 30-70, the items with the highest item-to-total correlations and factor loadings were selected for the MIDI. Next, forward regressions were computed to determine the smallest number of items needed to account for more than 90 percent of the total scale variance. For example, items on the conscientiousness scale included “organized,” “responsible,” “hardworking,” and “careless.” Response categories ranged from 1 (a lot) to 4 (not at all). Responses were reverse scored so that a higher score indicated higher indication of a given personality trait. All items were averaged to obtain a composite score for each personality trait.

*Childhood abuse*. Childhood abuse was assessed in the pre-baseline wave (at t_0_;2006/2008), rather than in the baseline wave with the other covariates, because this variable was not available in 2010/2012.

**Appendix Text 3**

**Proof Illustrating How Adjusting for Pre-Baseline Levels of Friendship Can Help Us Evaluate How “Change” in Friendship is Associated with Subsequent Health and Well-Being Outcomes Over Time**

Let Y be the outcome in 2014/2016, A_1_ the friendship exposure in 2010/2012, A_0_ the friendship exposure in 2006/2008, C the set of covariates in 2006/2008. For a continuous outcome, the regression model is: E[Y|a_0_, a_1_, c] = v + b_0_a_0_ b_1_a_1_+ b_2_’c

Let Y_a_ denote the potential outcome for Y for an individual under an intervention to set A_1_ to a. For an individual with baseline friendship exposure A_0_ = a_0_ and covariates c in 2006/2008, under the no-confounding (and positivity and consistency) and modeling assumptions, a change in friendship of d points A_0_ = a_0_ to A_1_ = a_0_ + d in 2010/2012, rather than maintaining friendship of A_1_ = a_0_ in 2010/2012, will give rise to an effect (a difference in potential outcomes for Y) of:

E[Y_a0+d_| A_0_ = a_0_, c] - E[Y_a0_| A_0_ = a_0_, c]

= E[Y_a0+d_| A_1_ = a_0_ + d, A_0_ = a_0_, c] - E[Y_a0_| A_1_ = a_0_, A_0_ = a_0_, c]

= E[Y| A_1_ = a_0_+d, A_0_ = a_0_, c] - E[Y| A_1_ = a_0_, A_0_ = a_0_, c]

= [v + b_0_a_0_ + b_1_(a_0_+d) + b_2_’c] - [v + b_0_a_0_ + b_1_a_0_ + b_2_’c]

= b_1_d

where the first equality follows by the no-confounding assumption, the second by consistency, and the third by the statistical model.

## Appendix Text References

1. Weir DR. Validating mortality ascertainment in the Health and Retirement Study. Published online 2016. https://hrs.isr.umich.edu/publications/biblio/9022

2. Fisher GG, Faul JD, Weir DR, Wallace RB. Documentation of chronic disease measures in the Heath and Retirement Study (HRS/AHEAD).Published 2005. https://hrs.isr.umich.edu/publications/biblio/5619

3. World Health Organization. Physical status: the use and interpretation of anthropometry: report of a WHO expert committee. Published online 1995. https://apps.who.int/iris/bitstream/handle/10665/37003/WHO_TRS_854.pdf

4. Fisher GG, Halimah H, Faul JD, Rogers WL, Weir DR. Health and Retirement Study imputation of cognitive functioning measures:1992 – 2014. Health and Retirement Study. Published January 13, 2017. https://hrs.isr.umich.edu/sites/default/files/biblio/COGIMPdd.pdf

5. Ofstedal MB, Fisher GG, Herzog AR. Documentation of cognitive functioning measures in the Health and Retirement Study. Published 2005. http://hrsonline.isr.umich.edu/sitedocs/userg/dr-006.pdf

6. Crimmins EM, Kim JK, Langa KM, Weir DR. Assessment of cognition using surveys and neuropsychological assessment: the health and retirement study and the aging, demographics, and memory study. *J Gerontol B Psychol Sci Soc Sci*. 2011;66 Suppl 1:i162-171. doi:10.1093/geronb/gbr048

7. Langa KM, Plassman BL, Wallace RB, et al. The aging, demographics, and memory study: study design and methods. *Neuroepidemiology*. 2005;25(4):181-191. doi:10.1159/000087448

8. Drinking levels defined | National Institute on Alcohol Abuse and Alcoholism (NIAAA). Accessed March 31, 2019. https://www.niaaa.nih.gov/alcohol-health/overview-alcohol-consumption/moderate-binge-drinking

9. Nandi A, Glymour MM, Subramanian SV. Association among socioeconomic status, health behaviors, and all-cause mortality in the United States. *Epidemiol Camb Mass*. 2014;25(2):170-177. doi:10.1097/EDE.0000000000000038

10. Jenkins CD, Stanton B-A, Niemcryk SJ, Rose RM. A scale for the estimation of sleep problems in clinical research. *J Clin Epidemiol*. 1988;41(4):313-321. doi:10.1016/0895-4356(88)90138-2

11. Watson D, Clark LA. The PANAS-X: Manual for the positive and negative affect schedule-expanded form. Published online 1994. https://ir.uiowa.edu/cgi/viewcontent.cgi?article=1011&context=psychology_pubs

12. Diener E, Emmons RA, Larsen RJ, Griffin S. The satisfaction with life scale. *J Pers Assess*. 1985;49(1):71-75. doi:10.1207/s15327752jpa4901_13

13. Scheier MF, Carver CS, Bridges MW. Distinguishing optimism from neuroticism (and trait anxiety, self-mastery, and self-esteem): a reevaluation of the Life Orientation Test. *J Pers Soc Psychol*. 1994;67:1063-1078. doi:10.1037/0022-3514.67.6.1063

14. Ryff CD, Keyes CLM. The structure of psychological well-being revisited. *J Pers Soc Psychol*. 1995;69:719-727. doi:10.1037/0022-3514.69.4.719

15. Abbott R, Ploubidis G, Huppert F, Kuh D, Wadsworth M, Croudace T. Psychometric evaluation and predictive validity of Ryff’s psychological well-being items in a UK birth cohort sample of women. *Health Qual Life Outcomes*. 2006;4:76. doi:10.1186/1477-7525-4-76

16. Radloff LS. The CES-D Scale: A self-report depression scale for research in the general population. *Appl Psychol Meas*. 1977;1:385-401. doi:10.1177/014662167700100306

17. Steffeck D. Documentation of affective functioning measures in the Health and Retirement Study. Published online 2000. ttps://hrs.isr.umich.edu/sites/default/files/biblio/dr-005.pdf

18. Beck AT, Weissman A, Lester D, Trexler L. The measurement of pessimism: the hopelessness scale. *J Consult Clin Psychol*. 1974;42:861-865. doi:10.1037/h0037562

19. Everson SA, Kaplan GA, Goldberg DE, Salonen R, Salonen JT. Hopelessness and 4-year progression of carotid atherosclerosis: the Kuopio ischemic heart disease risk factor study. *Arterioscler Thromb Vasc Biol*. 1997;17:1490-1495. doi:10.1161/01.ATV.17.8.1490

20. Russell DW. UCLA Loneliness Scale (Version 3): reliability, validity, and factor structure. *J Pers Assess*. 1996;66(1):20-40. doi:10.1207/s15327752jpa6601_2

21. Teo AR, Choi H, Andrea SB, et al. Does mode of contact with different types of social relationships predict depression in older adults? Evidence from a nationally representative survey. *J Am Geriatr Soc*. 2015;63(10):2014-2022. doi:10.1111/jgs.13667

22. Lachman M, Weaver S. Midlife Development Inventory (MIDI) personality scales: scale construction and scoring (Technical Report). Waltham, MA: Brandeis University, Psychology Department. Published online 1997.

**Appendix Table 1. Change in Friendship from the Pre-Baseline Wave (t_0_) to the Baseline Wave (t_1_)^a,b^**

| **Pre-Baseline Wave (t_0_)** | **Baseline Wave (t_1_)** | | | |
| --- | --- | --- | --- | --- |
|  | Quartile 1 | Quartile 2 | Quartile 3 | Quartile 4 |
|  | % | % | % | % |
| Quartile 1 | 54.9 | 26.5 | 12.8 | 5.0 |
| Quartile 2 | 25.3 | 35.3 | 27.2 | 11.7 |
| Quartile 3 | 13.0 | 26.1 | 34.1 | 27.2 |
| Quartile 4 | 5.8 | 12.2 | 25.9 | 56.2 |

^a^The percent of people in quartile 1, 2, 3, or 4 in the pre-baseline wave (t_0_) who end up in a quartile 1, 2, 3, or 4 later in the baseline wave (t_1_).

^b^The values in the first row (quartile 1) do not add up to 100% because of rounding.

## Appendix Table 2. Friendship and Subsequent Health/Well-Being (Adjustment for Conventional/All Covariates: N=12,988)^a,b,c^

|  | **Friendship** | | | |
| --- | --- | --- | --- | --- |
| **Outcomes** | Quartile 1  (n=3,249) | Conventionally-Adjusted Models^d^  Quartile 4 | Fully-Adjusted Models^e^  Quartile 4 |  |
|  | (Reference) | RR/OR/β (95% CI) | RR/OR/β (95% CI) |  |
| **Physical Health** |  |  |  |  |
| All-cause mortality | 1.00 | 0.67 (0.56, 0.79)*** | 0.76 (0.62, 0.95)* |  |
| Number of chronic conditions | 0.00 | -0.14 (-0.19, 0.08)*** | -0.01 (-0.05, 0.02) |  |
| Diabetes^f^ | 1.00 | 0.88 (0.68, 1.14) | 0.90 (0.64, 1.28) |  |
| Hypertension^g^ | 1.00 | 1.10 (0.90, 1.25) | 1.05 (0.81, 1.37) |  |
| Stroke^h^ | 1.00 | 0.64 (0.45, 0.92)* | 0.67 (0.44, 1.01) |  |
| Cancer^i^ | 1.00 | 1.10 (0.85, 1.42) | 0.98 (0.73, 1.33) |  |
| Heart disease^j^ | 1.00 | 0.99 (0.80, 1.24) | 1.01 (0.77, 1.34) |  |
| Lung disease^k^ | 1.00 | 0.83 (0.62, 1.10) | 0.80 (0.55, 1.17) |  |
| Arthritis^l^ | 1.00 | 1.03 (0.84, 1.27) | 1.22 (0.96, 1.55) |  |
| Overweight/obesity^m^ | 1.00 | 0.89 (0.69, 1.15) | 1.00 (0.78, 1.33) |  |
| Physical functioning limitations^n^ | 1.00 | 0.71 (0.60, 0.83) | 0.87 (0.73, 1.05) |  |
| Cognitive impairment^o^ | 1.00 | 0.86 (0.73, 1.02) | 0.89 (0.72, 1.10) |  |
| Chronic pain^p^ | 1.00 | 0.80 (0.70, 0.93) | 0.93 (0.78, 1.12) |  |
| Self-rated health | 0.00 | 0.28 (0.23, 0.33)*** | 0.09 (0.04, 0.15)** |  |
| **Health Behaviors** |  |  |  |  |
| Heavy drinking | 1.00 | 1.38 (1.08, 1.77)* | 1.48 (0.96, 2.30) |  |
| Smoking | 1.00 | 0.82 (0.67, 1.00) | 1.43 (1.03, 1.99)* |  |
| Frequent physical activity | 1.00 | 1.19 (1.12, 1.28)*** | 1.09 (1.00, 1.18)* |  |
| Sleep problems | 1.00 | 0.86 (0.79, 0.93)*** | 1.05 (0.94, 1.17) |  |
| **Psychological Well-Being** |  |  |  |  |
| Positive affect | 0.00 | 0.63 (0.57, 0.88)*** | 0.22 (0.15, 0.28)** |  |
| Life satisfaction | 0.00 | 0.42 (0.35, 0.48)*** | 0.17 (0.10, 0.23)** |  |
| Optimism | 0.00 | 0.54 (0.46, 0.61)*** | 0.18 (0.09, 0.27)** |  |
| Purpose in life | 0.00 | 0.54 (0.49, 0.59)*** | 0.17 (0.12, 0.23)** |  |
| Mastery | 0.00 | 0.44 (0.38, 0.49)*** | 0.20 (0.14, 0.27)** |  |
| Health mastery | 0.00 | 0.26 (0.20, 0.32)*** | 0.11 (0.03, 0.19)* |  |
| Financial mastery | 0.00 | 0.30 (0.23, 0.38)*** | 0.15 (0.06, 0.23)** |  |

| **Psychological Distress** |  |  |  |
| --- | --- | --- | --- |
| Depression | 1.00 | 0.41 (0.44, 0.60)*** | 0.83 (0.68, 1.00)* |
| Depressive symptoms | 0.00 | -0.34 (-0.40,-0.29)*** | -0.09 (-0.15,-0.04)** |
| Hopelessness | 0.00 | -0.47 (-0.53,-0.41)*** | -0.14 (-0.21,-0.07)** |
| Negative affect | 0.00 | -0.48 (-0.54,-0.43)*** | -0.20 (-0.26,-0.13)** |
| Perceived constraints | 0.00 | -0.44 (-0.49,-0.39)*** | -0.15 (-0.21,-0.09)** |
| **Social Factors** |  |  |  |
| Loneliness | 0.00 | -0.54 (-0.61,-0.47)** | -0.21 (-0.29,-0.13)** |
| Not living with a spouse/partner | 1.00 | 1.13 (1.04, 1.23)* | 1.20 (1.09, 1.32)** |
| Contact children ≥1x/week | 1.00 | 1.09 (1.03, 1.17)** | 1.02 (0.95, 1.10) |
| Contact other family ≥1x/week | 1.00 | 1.34 (1.23, 1.45)*** | 1.18 (1.07, 1.31)** |
| Contact friends ≥1x/week | 1.00 | 2.53 (2.34, 2.74)*** | 1.96 (1.77, 2.17)** |

Statistical significance (*p<0.05 before Bonferroni correction;**p<0.01 before Bonferroni correction;***p<0.05 after Bonferroni correction (the p-value cutoff for Bonferroni correction is p = 0.05/35 outcomes = p<0.001)).

Abbreviations: CI, confidence interval; OR, odds ratio; RR, risk ratio.

^a^If the reference value is “1,” the effect estimate is OR or RR; if the reference value is “0,” the effect estimate is β.

^c^An outcome-wide analytic approach was used, and a separate model for each outcome was run. A different type of model was run depending on the nature of the outcome: 1) for each binary outcome with a prevalence of ≥10%, a generalized linear model (with a log link and Poisson distribution) was used to estimate a RR; 2) for each binary outcome with a prevalence of <10%, a logistic regression model was used to estimate an OR; and 3) for each continuous outcome, a linear regression model was used to estimate a β.

^c^All continuous outcomes were standardized (mean = 0; standard deviation = 1), and β was the standardized effect size.

^d^The analytic sample was restricted to those who had participated in the baseline wave (t_1_; 2010 or 2012). Multiple imputation was performed to impute missing data on the exposure, covariates, and outcomes. All models controlled for sociodemographic characteristics (age, sex, race/ethnicity, marital status, annual household income, total wealth, level of education). These variables were controlled for in the pre-baseline wave (t_0_; in 2006 or 2008).

^e^The analytic sample was restricted to those who had participated in the baseline wave (t_1_; 2010 or 2012). Multiple imputation was performed to impute missing data on the exposure, covariates, and outcomes. All models controlled for sociodemographic characteristics (age, sex, race/ethnicity, marital status, annual household income, total wealth, level of education, employment status, health insurance, geographic region), pre-baseline childhood abuse, pre-baseline religious service attendance, pre-baseline values of the outcome variables (diabetes, hypertension, stroke, cancer, heart disease, lung disease, arthritis, overweight/obesity, physical functioning limitations, cognitive impairment, chronic pain, self-rated health, binge drinking, current smoking status, physical activity, sleep problems, positive affect, life satisfaction, optimism, purpose in life, personal mastery, health mastery, financial mastery, depressive symptoms, hopelessness, negative affect, perceived constraints, loneliness, living with spouse/partner, contact children >1x/week, contact other family >1x/week, contact friends >1x/week), personality factors (openness, conscientiousness, extraversion, agreeableness, neuroticism) and the pre-baseline value of the exposure. These variables were controlled for in the wave pre-baseline to the exposure assessment (in t_0_; 2006 or 2008).

^f^includes only study participants with no history of diabetes (*N* = 10,043).

^g^includes only study participants with no history of hypertension (*N* = 5,162). For this analysis, we did not control for hypertension in wave 1 because the cell size was too small and the analysis did not converge.

^h^includes only study participants with no history of stroke (*N* = 11,924).

^i^includes only study participants with no history of cancer (*N* = 10,883).

^j^includes only study participants with no history of heart disease (*N* = 9,727).

^k^includes only study participants with no history of lung disease (*N* = 11,685).

^l^includes only study participants with no history of arthritis (*N* = 5,058). For this analysis, we did not control for arthritis in wave 1 because the cell size was too small and the analysis did not converge.

^m^includes only study participants who were not overweight/obese (*N* = 3,754).

^n^includes only study participants who did not have physical functioning limitations (*N* = 9,797).

^o^includes only study participants who did not have cognitive impairment (*N* = 10,408).

^p^includes only study participants who did not have chronic pain (*N* = 8,288).

Appendix Table 3. Friendship Network Size and Subsequent Health and Well-being (Health and Retirement Study [HRS]:

N=12,998)^a,b,c,d^

| **Outcomes** | **Friendship Network Size** | | | |  |
| --- | --- | --- | --- | --- | --- |
|  | Quartile 1  (n=5,685) | Quartile 2  (n=1,952) | Quartile 3  (n=2,612) | Quartile 4  (n=2,739) | |
|  | (Reference) | RR/OR/β (95% CI) | RR/OR/β (95% CI) | RR/OR/β (95% CI) | |
| **Physical Health** |  |  |  |  | |
| All-cause mortality | 1.00 | 0.96 (0.81, 1.13) | 1.00 (0.86, 1.16) | 0.92 (0.78, 1.08) | |
| Number of chronic conditions | 0.00 | -0.00 (-0.04, 0.03) | 0.01 (-0.02, 0.05) | 0.01 (-0.03, 0.04) | |
| Diabetes | 1.00 | 0.97 (0.87, 1.08) | 1.03 (0.94, 1.14) | 1.00 (0.91, 1.11) | |
| Hypertension | 1.00 | 0.99 (0.93, 1.06) | 1.01 (0.95, 1.07) | 1.01 (0.95, 1.08) | |
| Stroke | 1.00 | 0.88 (0.75, 1.05) | 1.00 (0.87, 1.15) | 0.94 (0.81, 1.09) | |
| Cancer | 1.00 | 1.03 (0.92, 1.16) | 1.07 (0.96, 1.20) | 1.05 (0.93, 1.19) | |
| Heart disease | 1.00 | 1.01 (0.92, 1.12) | 0.99 (0.90, 1.08) | 1.00 (0.91, 1.10) | |
| Lung disease | 1.00 | 0.96 (0.82, 1.12) | 0.99 (0.86, 1.15) | 1.05 (0.92, 1.21) | |
| Arthritis | 1.00 | 1.02 (0.96, 1.09) | 1.00 (0.95, 1.07) | 0.99 (0.93, 1.05) | |
| Overweight/obesity | 1.00 | 0.99 (0.93, 1.06) | 0.99 (0.93, 1.05) | 1.00 (0.94, 1.06) | |
| Physical functioning limitations | 1.00 | 0.96 (0.86, 1.07) | 1.00 (0.91, 1.10) | 0.95 (0.86, 1.05) | |
| Cognitive impairment | 1.00 | 0.93 (0.83, 1.04) | 0.97 (0.88, 1.07) | 0.99 (0.89, 1.10) | |
| Chronic pain | 1.00 | 1.01 (0.93, 1.11) | 1.02 (0.94, 1.11) | 0.98 (0.90, 1.06) | |
| Self-rated health | 0.00 | 0.02 (-0.03, 0.07) | 0.01 (-0.04, 0.06) | 0.07 (0.02, 0.11)** | |
| **Health Behaviors** |  |  |  |  | |
| Heavy drinking | 1.00 | 1.01 (0.80, 1.27) | 1.19 (0.86, 1.63) | 1.22 (0.95, 1.55) | |
| Smoking | 1.00 | 1.04 (0.76, 1.43) | 0.89 (0.63, 1.27) | 0.97 (0.68, 1.39) | |
| Frequent physical activity | 1.00 | 1.02 (0.95, 1.09) | 1.04 (0.97, 1.10) | 1.06 (0.99, 1.13) | |
| Sleep problems | 1.00 | 1.01 (0.93, 1.10) | 1.03 (0.96, 1.12) | 0.99 (0.91, 1.07) | |
| **Psychological Well-Being** |  |  |  |  | |
| Positive affect | 0.00 | 0.04 (-0.01, 0.08) | 0.07 (0.03, 0.11)** | 0.11 (0.06, 0.16)** | |
| Life satisfaction | 0.00 | 0.03 (-0.04, 0.10) | 0.03 (-0.03, 0.09) | 0.10 (0.02, 0.18)* | |
| Optimism | 0.00 | 0.01 (-0.04, 0.05) | 0.04 (-0.01, 0.09) | 0.06 (0.01, 0.11)* | |
| Purpose in life | 0.00 | 0.02 (-0.03, 0.07) | 0.04 (-0.02, 0.09) | 0.08 (0.03, 0.13)** | |
| Mastery | 0.00 | -0.00 (-0.06, 0.05) | 0.01 (-0.06, 0.07) | 0.07 (0.02, 0.13)* | |
| Health mastery | 0.00 | -0.01 (-0.07, 0.05) | 0.00 (-0.05, 0.05) | 0.01 (-0.05, 0.08) | |
| Financial mastery | 0.00 | 0.01 (-0.06, 0.08) | 0.00 (-0.05, 0.05) | 0.02 (-0.04, 0.07) | |
| **Psychological Distress** |  |  |  |  | |
| Depression | 1.00 | 1.01 (0.86, 1.19) | 0.87 (0.75, 1.01) | 0.94 (0.77, 1.16) | |
| Depressive symptoms | 0.00 | 0.01 (-0.04, 0.07) | -0.04 (-0.08, 0.00) | -0.03 (-0.07, 0.02) | |
| Hopelessness | 0.00 | -0.03 (-0.08, 0.01) | -0.03 (-0.08, 0.02) | -0.05 (-0.10, -0.01)* | |
| Negative affect | 0.00 | -0.01 (-0.06, 0.03) | -0.03 (-0.08, 0.03) | -0.04 (-0.10, 0.01) | |
| Perceived constraints | 0.00 | -0.02 (-0.06, 0.03) | -0.02 (-0.06, 0.03) | -0.03 (-0.08, 0.02) | |
| **Social Factors** |  |  |  |  | |
| Loneliness | 0.00 | -0.04 (-0.09, 0.02) | -0.09 (-0.13, -0.04)** | -0.13 (-0.18, -0.09)** | |
| Not living with a spouse/partner | 1.00 | 1.02 (0.93, 1.10) | 1.02 (0.93, 1.11) | 1.04 (0.95, 1.13) | |
| Contact children ≥1x/week | 1.00 | 1.02 (0.96, 1.09) | 1.01 (0.95, 1.07) | 1.01 (0.95, 1.07) | |
| Contact other family ≥1x/week | 1.00 | 1.06 (0.98, 1.14) | 1.07 (0.99, 1.16) | 1.04 (0.96, 1.13) | |
| Contact friends ≥1x/week | 1.00 | 1.21 (1.13, 1.30)** | 1.28 (1.20, 1.36)** | 1.28 (1.21, 1.37)** | |

Abbreviations: CI, confidence interval; OR, odds ratio; RR, risk ratio.

^a^If the reference value is “1,” the effect estimate is OR or RR; if the reference value is “0,” the effect estimate is β.

^b^The analytic sample was restricted to those who had participated in the baseline wave (t_1_;2010 or 2012). Multiple imputation was performed to impute missing data on the exposure, covariates, and outcomes. All models controlled for sociodemographic characteristics (age, sex, race/ethnicity, marital status, annual household income, total wealth, level of education, employment status, health insurance, geographic region), pre-baseline childhood abuse, pre-baseline religious service attendance, pre-baseline values of the outcome variables (diabetes, hypertension, stroke, cancer, heart disease, lung disease, arthritis, overweight/obesity, physical functioning limitations, cognitive impairment, chronic pain, self-rated health, binge drinking, current smoking status, physical activity, sleep problems, positive affect, life satisfaction, optimism, purpose in life, mastery, health mastery, financial mastery, depressive symptoms, hopelessness, negative affect, perceived constraints, loneliness, living with spouse/partner, contact children <1x/week , contact other family <1x/week , contact friends <1x/week ), personality factors (openness, conscientiousness, extraversion, agreeableness, neuroticism) and the pre-baseline value of the exposure. These variables were controlled for in the pre-baseline was (in t0;2006 or 2008).

^c^We used an outcome-wide analytic approach and ran a separate model for each outcome. We ran a different type of model depending on the nature of the outcome: 1) for each binary outcome with a prevalence of ≥10%, we used a generalized linear model (with a log link and Poisson distribution) to estimate a RR; 2) for each binary outcome with a prevalence of <10%, we used a logistic regression model to estimate an OR; and 3) for each continuous outcome, we used a linear regression model to estimate a β.

^d^All continuous outcomes were standardized (mean=0; standard deviation=1), and β was the standardized effect size.

*p<0.05 before Bonferroni correction;**p<0.01 before Bonferroni correction;***p<0.05 after Bonferroni correction (the p-value cutoff for Bonferroni correction is p=0.05/35 outcomes=p<0.001).

Appendix Table 4. Friendship Network Contact Frequency (In-Person Meetups) and Subsequent Health and Well-being (Health and Retirement Study [HRS]:

N=12,998)^a,b,c,d^

| **Outcomes** | **Friendship Network Contact Frequency (In-Person Meetup Frequency)** | | | |  |
| --- | --- | --- | --- | --- | --- |
|  | Quartile 1  (n=3,785) | Quartile 2  (n=3,612) | Quartile 3  (n=3,916) | Quartile 4  (n=1,675) | |
|  | (Reference) | RR/OR/β (95% CI) | RR/OR/β (95% CI) | RR/OR/β (95% CI) | |
| **Physical Health** |  |  |  |  | |
| All-cause mortality | 1.00 | 0.91 (0.78, 1.06) | 0.81 (0.70, 0.95)** | 0.74 (0.61, 0.91)** | |
| Number of chronic conditions | 0.00 | 0.01 (-0.03, 0.04) | -0.00 (-0.04, 0.03) | -0.02 (-0.06, 0.02) | |
| Diabetes | 1.00 | 1.00 (0.92, 1.10) | 0.99 (0.90, 1.08) | 1.01 (0.89, 1.14) | |
| Hypertension | 1.00 | 1.00 (0.94, 1.06) | 1.01 (0.95, 1.07) | 1.00 (0.92, 1.08) | |
| Stroke | 1.00 | 0.91 (0.79, 1.05) | 0.88 (0.75, 1.04) | 0.86 (0.71, 1.04) | |
| Cancer | 1.00 | 1.09 (0.98, 1.22) | 1.06 (0.95, 1.19) | 1.03 (0.89, 1.19) | |
| Heart disease | 1.00 | 1.00 (0.91, 1.09) | 1.01 (0.92, 1.11) | 0.97 (0.86, 1.10) | |
| Lung disease | 1.00 | 1.03 (0.91, 1.18) | 0.98 (0.85, 1.12) | 0.91 (0.75, 1.10) | |
| Arthritis | 1.00 | 0.99 (0.94, 1.05) | 1.00 (0.94, 1.06) | 1.00 (0.93, 1.08) | |
| Overweight/obesity | 1.00 | 1.01 (0.95, 1.07) | 1.01 (0.95, 1.07) | 1.01 (0.93, 1.09) | |
| Physical functioning limitations | 1.00 | 0.94 (0.86, 1.03) | 0.94 (0.85, 1.02) | 0.91 (0.80, 1.03) | |
| Cognitive impairment | 1.00 | 0.93 (0.82, 1.05) | 0.94 (0.84, 1.05) | 0.93 (0.82, 1.05) | |
| Chronic pain | 1.00 | 0.96 (0.89, 1.04) | 1.00 (0.92, 1.09) | 0.97 (0.88, 1.08) | |
| Self-rated health | 0.00 | 0.05 (-0.00, 0.09) | 0.06 (0.00, 0.12)* | 0.12 (0.05, 0.18)** | |
| **Health Behaviors** |  |  |  |  | |
| Heavy drinking | 1.00 | 1.29 (1.03, 1.61)* | 1.42 (0.98, 2.05) | 1.31 (0.84, 2.03) | |
| Smoking | 1.00 | 1.25 (0.89, 1.76) | 1.47 (1.13, 1.92)** | 1.48 (0.99, 2.21) | |
| Frequent physical activity | 1.00 | 1.05 (0.98, 1.12) | 1.09 (1.01, 1.16)* | 1.10 (1.01, 1.20)* | |
| Sleep problems | 1.00 | 0.97 (0.89, 1.05) | 1.03 (0.95, 1.12) | 1.00 (0.90, 1.11) | |
| **Psychological Well-Being** |  |  |  |  | |
| Positive affect | 0.00 | 0.06 (0.02, 0.11)** | 0.10 (0.05, 0.15)** | 0.17 (0.10, 0.24)** | |
| Life satisfaction | 0.00 | 0.05 (0.01, 0.10)* | 0.08 (0.02, 0.13)** | 0.15 (0.07, 0.23)** | |
| Optimism | 0.00 | 0.03 (-0.03, 0.09) | 0.05 (-0.02, 0.11) | 0.10 (0.01, 0.18)* | |
| Purpose in life | 0.00 | 0.07 (-0.00, 0.13) | 0.07 (0.03, 0.12)** | 0.14 (0.07, 0.21)** | |
| Mastery | 0.00 | 0.07 (0.01, 0.13)* | 0.07 (-0.02, 0.16) | 0.16 (0.07, 0.25)** | |
| Health mastery | 0.00 | 0.04 (-0.01, 0.08) | 0.05 (0.00, 0.10)* | 0.08 (0.02, 0.15)* | |
| Financial mastery | 0.00 | 0.09 (0.03, 0.15)** | 0.10 (0.03, 0.16)** | 0.14 (0.08, 0.21)** | |
| **Psychological Distress** |  |  |  |  | |
| Depression | 1.00 | 0.87 (0.74, 1.01) | 0.85 (0.74, 0.99)* | 0.77 (0.63, 0.95)* | |
| Depressive symptoms | 0.00 | -0.08 (-0.12, -0.04)** | -0.08 (-0.12, -0.03)** | -0.13 (-0.19, -0.07)** | |
| Hopelessness | 0.00 | -0.06 (-0.11, -0.01)* | -0.05 (-0.12, 0.02) | -0.08 (-0.15, -0.01)* | |
| Negative affect | 0.00 | -0.08 (-0.13, -0.03)** | -0.09 (-0.13, -0.05)** | -0.18 (-0.25, -0.12)** | |
| Perceived constraints | 0.00 | -0.06 (-0.11, -0.01)* | -0.09 (-0.13, -0.05)** | -0.11 (-0.17, -0.05)** | |
| **Social Factors** |  |  |  |  | |
| Loneliness | 0.00 | -0.09 (-0.14, -0.04)** | -0.10 (-0.16, -0.05)** | -0.20 (-0.28, -0.11)** | |
| Not living with a spouse/partner | 1.00 | 1.01 (0.93, 1.10) | 1.08 (0.99, 1.17) | 1.13 (1.02, 1.25)* | |
| Contact children ≥1x/week | 1.00 | 1.02 (0.96, 1.08) | 1.02 (0.96, 1.08) | 1.02 (0.94, 1.10) | |
| Contact other family ≥1x/week | 1.00 | 1.10 (1.02, 1.17)** | 1.11 (1.03, 1.20)* | 1.13 (1.02, 1.25)* | |
| Contact friends ≥1x/week | 1.00 | 1.40 (1.29, 1.52)** | 1.66 (1.53, 1.81)** | 1.78 (1.60, 1.96)** | |

Abbreviations: CI, confidence interval; OR, odds ratio; RR, risk ratio.

^a^If the reference value is “1,” the effect estimate is OR or RR; if the reference value is “0,” the effect estimate is β.

^b^The analytic sample was restricted to those who had participated in the baseline wave (t_1_;2010 or 2012). Multiple imputation was performed to impute missing data on the exposure, covariates, and outcomes. All models controlled for sociodemographic characteristics (age, sex, race/ethnicity, marital status, annual household income, total wealth, level of education, employment status, health insurance, geographic region), pre-baseline childhood abuse, pre-baseline religious service attendance, pre-baseline values of the outcome variables (diabetes, hypertension, stroke, cancer, heart disease, lung disease, arthritis, overweight/obesity, physical functioning limitations, cognitive impairment, chronic pain, self-rated health, binge drinking, current smoking status, physical activity, sleep problems, positive affect, life satisfaction, optimism, purpose in life, mastery, health mastery, financial mastery, depressive symptoms, hopelessness, negative affect, perceived constraints, loneliness, living with spouse/partner, contact children <1x/week , contact other family <1x/week , contact friends <1x/week ), personality factors (openness, conscientiousness, extraversion, agreeableness, neuroticism) and the pre-baseline value of the exposure. These variables were controlled for in the pre-baseline was (in t0;2006 or 2008).

^c^We used an outcome-wide analytic approach and ran a separate model for each outcome. We ran a different type of model depending on the nature of the outcome: 1) for each binary outcome with a prevalence of ≥10%, we used a generalized linear model (with a log link and Poisson distribution) to estimate a RR; 2) for each binary outcome with a prevalence of <10%, we used a logistic regression model to estimate an OR; and 3) for each continuous outcome, we used a linear regression model to estimate a β.

^d^All continuous outcomes were standardized (mean=0; standard deviation=1), and β was the standardized effect size.

*p<0.05 before Bonferroni correction;**p<0.01 before Bonferroni correction;***p<0.05 after Bonferroni correction (the p-value cutoff for Bonferroni correction is p=0.05/35 outcomes=p<0.001).

Appendix Table 5. Friendship Network Contact Frequency (Calls) and Subsequent Health and Well-being (Health and Retirement Study [HRS]: N=12,998)^a,b,c,d^

| **Outcome** | **Friendship Network Contact Frequency (Calls)** | | |
| --- | --- | --- | --- |
|  | Tertile 1  (n=5,750) | Tertile 2  (n=4,260) | Tertile 3  (n=2,978) |
|  | (Reference) | RR/OR/β (95% CI) | RR/OR/β (95% CI) |
| **Physical Health** |  |  |  |
| All-cause mortality | 1.00 | 0.98 (0.85, 1.12) | 0.92 (0.77, 1.09) |
| Number of chronic conditions | 0.00 | 0.01 (-0.02, 0.04) | 0.00 (-0.03, 0.04) |
| Diabetes | 1.00 | 0.98 (0.90, 1.07) | 0.98 (0.89, 1.09) |
| Hypertension | 1.00 | 1.02 (0.97, 1.07) | 1.04 (0.98, 1.11) |
| Stroke | 1.00 | 1.01 (0.87, 1.17) | 0.93 (0.80, 1.09) |
| Cancer | 1.00 | 1.06 (0.96, 1.17) | 1.00 (0.88, 1.13) |
| Heart disease | 1.00 | 0.98 (0.90, 1.06) | 1.03 (0.93, 1.13) |
| Lung disease | 1.00 | 1.02 (0.89, 1.17) | 1.06 (0.90, 1.24) |
| Arthritis | 1.00 | 0.99 (0.94, 1.05) | 1.00 (0.94, 1.06) |
| Overweight/obesity | 1.00 | 1.00 (0.95, 1.06) | 0.98 (0.92, 1.04) |
| Physical functioning limitations | 1.00 | 0.93 (0.84, 1.02) | 1.02 (0.92, 1.12) |
| Cognitive impairment | 1.00 | 0.95 (0.86, 1.06) | 1.07 (0.96, 1.19) |
| Chronic pain | 1.00 | 0.99 (0.92, 1.06) | 1.00 (0.92, 1.09) |
| Self-rated health | 0.00 | 0.00 (-0.03, 0.04) | 0.00 (-0.05, 0.05) |
| **Health Behaviors** |  |  |  |
| Heavy drinking | 1.00 | 1.19 (0.92, 1.55) | 1.06 (0.85, 1.33) |
| Smoking | 1.00 | 1.12 (0.87, 1.46) | 1.33 (0.99, 1.80) |
| Frequent physical activity | 1.00 | 1.03 (0.97, 1.09) | 1.01 (0.94, 1.10) |
| Sleep problems | 1.00 | 1.00 (0.93, 1.07) | 1.05 (0.97, 1.14) |
| **Psychological Well-Being** |  |  |  |
| Positive affect | 0.00 | 0.07 (0.01, 0.12)* | 0.06 (-0.01, 0.12) |
| Life satisfaction | 0.00 | 0.04 (-0.02, 0.09) | 0.03 (-0.02, 0.08) |
| Optimism | 0.00 | 0.02 (-0.03, 0.06) | -0.00 (-0.06, 0.06) |
| Purpose in life | 0.00 | 0.09 (0.04, 0.14)** | 0.10 (0.05, 0.16)** |
| Mastery | 0.00 | 0.04 (-0.02, 0.10) | 0.07 (-0.01, 0.14) |
| Health mastery | 0.00 | 0.01 (-0.04, 0.07) | 0.02 (-0.04, 0.08) |
| Financial mastery | 0.00 | 0.02 (-0.03, 0.07) | 0.03 (-0.02, 0.08) |
| **Psychological Distress** |  |  |  |
| Depression | 1.00 | 0.92 (0.80, 1.05) | 0.97 (0.84, 1.12) |
| Depressive symptoms | 0.00 | -0.02 (-0.06, 0.02) | -0.01 (-0.06, 0.04) |
| Hopelessness | 0.00 | -0.04 (-0.08, 0.00) | 0.00 (-0.06, 0.06) |
| Negative affect | 0.00 | -0.04 (-0.08, 0.01) | -0.05 (-0.10, 0.00) |
| Perceived constraints | 0.00 | -0.02 (-0.06, 0.02) | 0.01 (-0.04, 0.06) |
| **Social Factors** |  |  |  |
| Loneliness | 0.00 | -0.06 (-0.13, -0.00)* | -0.08 (-0.16, 0.01) |
| Not living with a spouse/partner | 1.00 | 1.09 (1.01, 1.17)* | 1.18 (1.09, 1.27)** |
| Contact children ≥1x/week | 1.00 | 1.02 (0.96, 1.07) | 1.04 (0.97, 1.11) |
| Contact other family ≥1x/week | 1.00 | 1.13 (1.05, 1.21)** | 1.18 (1.09, 1.28)** |
| Contact friends ≥1x/week | 1.00 | 1.46 (1.38, 1.55)** | 1.59 (1.47, 1.72)** |

Abbreviations: CI, confidence interval; OR, odds ratio; RR, risk ratio.

^a^If the reference value is “1,” the effect estimate is OR or RR; if the reference value is “0,” the effect estimate is β.

^b^The analytic sample was restricted to those who had participated in the baseline wave (t_1_;2010 or 2012). Multiple imputation was performed to impute missing data on the exposure, covariates, and outcomes. All models controlled for sociodemographic characteristics (age, sex, race/ethnicity, marital status, annual household income, total wealth, level of education, employment status, health insurance, geographic region), pre-baseline childhood abuse, pre-baseline religious service attendance, pre-baseline values of the outcome variables (diabetes, hypertension, stroke, cancer, heart disease, lung disease, arthritis, overweight/obesity, physical functioning limitations, cognitive impairment, chronic pain, self-rated health, binge drinking, current smoking status, physical activity, sleep problems, positive affect, life satisfaction, optimism, purpose in life, mastery, health mastery, financial mastery, depressive symptoms, hopelessness, negative affect, perceived constraints, loneliness, living with spouse/partner, contact children <1x/week , contact other family <1x/week , contact friends <1x/week ), personality factors (openness, conscientiousness, extraversion, agreeableness, neuroticism) and the pre-baseline value of the exposure. These variables were controlled for in the pre-baseline was (in t0;2006 or 2008).

^c^We used an outcome-wide analytic approach and ran a separate model for each outcome. We ran a different type of model depending on the nature of the outcome: 1) for each binary outcome with a prevalence of ≥10%, we used a generalized linear model (with a log link and Poisson distribution) to estimate a RR; 2) for each binary outcome with a prevalence of <10%, we used a logistic regression model to estimate an OR; and 3) for each continuous outcome, we used a linear regression model to estimate a β.

^d^All continuous outcomes were standardized (mean=0; standard deviation=1), and β was the standardized effect size.

*p<0.05 before Bonferroni correction;**p<0.01 before Bonferroni correction;***p<0.05 after Bonferroni correction (the p-value cutoff for Bonferroni correction is p=0.05/35 outcomes=p<0.001).

Appendix Table 6. Friendship Network Contact Frequency (Writing) and Subsequent Health and Well-being (Health and Retirement Study [HRS]: N=12,998)^a,b,c,d^

| **Outcome** | **Friendship Network Contact Frequency (Writing)** | | |
| --- | --- | --- | --- |
|  | Tertile 1  (n=8,240) | Tertile 2  (n=1,783) | Tertile 3  (n=2,965) |
|  | (Reference) | RR/OR/β (95% CI) | RR/OR/β (95% CI) |
| **Physical Health** |  |  |  |
| All-cause mortality | 1.00 | 1.06 (0.86, 1.31) | 0.90 (0.71, 1.14) |
| Number of chronic conditions | 0.00 | -0.02 (-0.06, 0.02) | 0.01 (-0.03, 0.04) |
| Diabetes | 1.00 | 0.97 (0.83, 1.12) | 1.02 (0.90, 1.17) |
| Hypertension | 1.00 | 0.97 (0.90, 1.04) | 0.99 (0.93, 1.06) |
| Stroke | 1.00 | 0.79 (0.63, 0.99)* | 0.90 (0.70, 1.15) |
| Cancer | 1.00 | 1.02 (0.89, 1.16) | 1.04 (0.91, 1.18) |
| Heart disease | 1.00 | 1.00 (0.89, 1.12) | 0.96 (0.87, 1.07) |
| Lung disease | 1.00 | 1.05 (0.88, 1.24) | 1.08 (0.91, 1.28) |
| Arthritis | 1.00 | 1.01 (0.94, 1.08) | 1.00 (0.93, 1.07) |
| Overweight/obesity | 1.00 | 1.01 (0.94, 1.08) | 1.02 (0.96, 1.09) |
| Physical functioning limitations | 1.00 | 0.88 (0.78, 1.00)* | 0.94 (0.83, 1.08) |
| Cognitive impairment | 1.00 | 0.78 (0.66, 0.92)** | 0.78 (0.65, 0.93)** |
| Chronic pain | 1.00 | 0.99 (0.90, 1.09) | 1.02 (0.93, 1.13) |
| Self-rated health | 0.00 | 0.07 (0.02, 0.11)** | 0.07 (0.02, 0.12)** |
| **Health Behaviors** |  |  |  |
| Heavy drinking | 1.00 | 1.12 (0.79, 1.59) | 1.02 (0.74, 1.42) |
| Smoking | 1.00 | 0.87 (0.61, 1.25) | 0.98 (0.63, 1.52) |
| Frequent physical activity | 1.00 | 1.10 (1.03, 1.18)** | 1.08 (1.01, 1.16)* |
| Sleep problems | 1.00 | 0.99 (0.90, 1.10) | 1.00 (0.92, 1.10) |
| **Psychological Well-Being** |  |  |  |
| Positive affect | 0.00 | 0.09 (0.04, 0.15)** | 0.13 (0.08, 0.19)** |
| Life satisfaction | 0.00 | 0.08 (0.01, 0.14)* | 0.11 (0.05, 0.16)** |
| Optimism | 0.00 | 0.05 (-0.00, 0.10) | 0.11 (0.03, 0.18)** |
| Purpose in life | 0.00 | 0.06 (0.02, 0.11)** | 0.07 (0.03, 0.12)** |
| Mastery | 0.00 | 0.04 (-0.01, 0.09) | 0.10 (0.04, 0.16)** |
| Health mastery | 0.00 | 0.05 (-0.00, 0.11) | 0.07 (0.00, 0.13)* |
| Financial mastery | 0.00 | 0.06 (-0.01, 0.13) | 0.04 (-0.01, 0.09) |
| **Psychological Distress** |  |  |  |
| Depression | 1.00 | 1.03 (0.81, 1.31) | 0.93 (0.75, 1.14) |
| Depressive symptoms | 0.00 | -0.02 (-0.07, 0.03) | -0.03 (-0.08, 0.03) |
| Hopelessness | 0.00 | -0.06 (-0.11, -0.01)* | -0.08 (-0.15, -0.01)* |
| Negative affect | 0.00 | 0.01 (-0.05, 0.06) | -0.02 (-0.08, 0.04) |
| Perceived constraints | 0.00 | -0.06 (-0.11, -0.00)* | -0.08 (-0.15, -0.00)* |
| **Social Factors** |  |  |  |
| Loneliness | 0.00 | -0.01 (-0.09, 0.07) | -0.01 (-0.08, 0.05) |
| Not living with a spouse/partner | 1.00 | 1.09 (1.01, 1.17)* | 1.03 (0.93, 1.14) |
| Contact children ≥1x/week | 1.00 | 1.02 (0.96, 1.07) | 1.02 (0.95, 1.09) |
| Contact other family ≥1x/week | 1.00 | 1.13 (1.05, 1.21)** | 1.10 (1.02, 1.20)* |
| Contact friends ≥1x/week | 1.00 | 1.46 (1.38, 1.55)** | 1.24 (1.14, 1.34)** |

Abbreviations: CI, confidence interval; OR, odds ratio; RR, risk ratio.

^a^If the reference value is “1,” the effect estimate is OR or RR; if the reference value is “0,” the effect estimate is β.

^b^The analytic sample was restricted to those who had participated in the baseline wave (t_1_;2010 or 2012). Multiple imputation was performed to impute missing data on the exposure, covariates, and outcomes. All models controlled for sociodemographic characteristics (age, sex, race/ethnicity, marital status, annual household income, total wealth, level of education, employment status, health insurance, geographic region), pre-baseline childhood abuse, pre-baseline religious service attendance, pre-baseline values of the outcome variables (diabetes, hypertension, stroke, cancer, heart disease, lung disease, arthritis, overweight/obesity, physical functioning limitations, cognitive impairment, chronic pain, self-rated health, binge drinking, current smoking status, physical activity, sleep problems, positive affect, life satisfaction, optimism, purpose in life, mastery, health mastery, financial mastery, depressive symptoms, hopelessness, negative affect, perceived constraints, loneliness, living with spouse/partner, contact children <1x/week , contact other family <1x/week , contact friends <1x/week ), personality factors (openness, conscientiousness, extraversion, agreeableness, neuroticism) and the pre-baseline value of the exposure. These variables were controlled for in the pre-baseline was (in t0;2006 or 2008).

^c^We used an outcome-wide analytic approach and ran a separate model for each outcome. We ran a different type of model depending on the nature of the outcome: 1) for each binary outcome with a prevalence of ≥10%, we used a generalized linear model (with a log link and Poisson distribution) to estimate a RR; 2) for each binary outcome with a prevalence of <10%, we used a logistic regression model to estimate an OR; and 3) for each continuous outcome, we used a linear regression model to estimate a β.

^d^All continuous outcomes were standardized (mean=0; standard deviation=1), and β was the standardized effect size.

*p<0.05 before Bonferroni correction;**p<0.01 before Bonferroni correction;***p<0.05 after Bonferroni correction (the p-value cutoff for Bonferroni correction is p=0.05/35 outcomes=p<0.001).

Appendix Table 7. Friendship Network Quality (Positive Social Support from Friends) and Subsequent Health and Well-being (Health and Retirement Study [HRS]: N=12,998)^a,b,c,d^

| **Outcomes** | **Friendship Network Quality (Positive Social Support from Friends)** | | | |  |
| --- | --- | --- | --- | --- | --- |
|  | Quartile 1  (n=3,247) | Quartile 2  (n=3,814) | Quartile 3  (n=3,394) | Quartile 4  (n=2,533) | |
|  | (Reference) | RR/OR/β (95% CI) | RR/OR/β (95% CI) | RR/OR/β (95% CI) | |
| **Physical Health** |  |  |  |  | |
| All-cause mortality | 1.00 | 0.98 (0.83, 1.14) | 0.91 (0.75, 1.10) | 0.96 (0.77, 1.21) | |
| Number of chronic conditions | 0.00 | -0.02 (-0.05, 0.01) | -0.03 (-0.07, 0.00) | -0.04 (-0.08, -0.00)* | |
| Diabetes | 1.00 | 1.00 (0.90, 1.10) | 0.94 (0.85, 1.05) | 0.94 (0.83, 1.06) | |
| Hypertension | 1.00 | 1.00 (0.94, 1.06) | 1.00 (0.93, 1.07) | 1.00 (0.92, 1.08) | |
| Stroke | 1.00 | 0.96 (0.82, 1.12) | 0.87 (0.74, 1.01) | 0.88 (0.73, 1.06) | |
| Cancer | 1.00 | 1.03 (0.92, 1.15) | 1.07 (0.95, 1.21) | 1.09 (0.94, 1.26) | |
| Heart disease | 1.00 | 0.96 (0.88, 1.05) | 0.98 (0.89, 1.08) | 0.97 (0.86, 1.09) | |
| Lung disease | 1.00 | 1.10 (0.94, 1.29) | 1.05 (0.86, 1.27) | 1.02 (0.85, 1.24) | |
| Arthritis | 1.00 | 0.99 (0.93, 1.05) | 1.01 (0.94, 1.08) | 0.99 (0.91, 1.06) | |
| Overweight/obesity | 1.00 | 0.97 (0.91, 1.03) | 0.95 (0.89, 1.01) | 0.94 (0.87, 1.01) | |
| Physical functioning limitations | 1.00 | 0.95 (0.84, 1.07) | 0.98 (0.88, 1.10) | 0.96 (0.84, 1.10) | |
| Cognitive impairment | 1.00 | 0.97 (0.88, 1.08) | 1.00 (0.89, 1.11) | 1.01 (0.88, 1.17) | |
| Chronic pain | 1.00 | 0.95 (0.88, 1.03) | 0.98 (0.90, 1.08) | 0.98 (0.88, 1.09) | |
| Self-rated health | 0.00 | -0.02 (-0.07, 0.03) | -0.03 (-0.08, 0.02) | -0.01 (-0.07, 0.06) | |
| **Health Behaviors** |  |  |  |  | |
| Heavy drinking | 1.00 | 1.29 (0.95, 1.75) | 1.09 (0.79, 1.49) | 1.39 (0.86, 2.24) | |
| Smoking | 1.00 | 1.20 (0.89, 1.62) | 1.27 (0.93, 1.72) | 1.54 (1.00, 2.36) | |
| Frequent physical activity | 1.00 | 1.02 (0.96, 1.09) | 1.02 (0.95, 1.09) | 1.02 (0.94, 1.11) | |
| Sleep problems | 1.00 | 0.96 (0.88, 1.06) | 1.02 (0.90, 1.14) | 1.03 (0.89, 1.18) | |
| **Psychological Well-Being** |  |  |  |  | |
| Positive affect | 0.00 | 0.08 (0.03, 0.14)** | 0.13 (0.07, 0.20)** | 0.22 (0.16, 0.29)** | |
| Life satisfaction | 0.00 | 0.05 (-0.01, 0.11) | 0.07 (-0.02, 0.15) | 0.13 (0.01, 0.25)* | |
| Optimism | 0.00 | 0.05 (0.00, 0.10)* | 0.11 (0.06, 0.17)** | 0.17 (0.09, 0.24)** | |
| Purpose in life | 0.00 | 0.06 (0.02, 0.11)** | 0.08 (0.02, 0.14)* | 0.12 (0.04, 0.19)** | |
| Mastery | 0.00 | 0.08 (0.03, 0.13)** | 0.10 (0.05, 0.15)** | 0.20 (0.14, 0.26)** | |
| Health mastery | 0.00 | 0.03 (-0.02, 0.07) | 0.06 (0.01, 0.11)* | 0.09 (0.02, 0.16)* | |
| Financial mastery | 0.00 | 0.06 (0.01, 0.11)* | 0.09 (0.03, 0.14)** | 0.12 (0.07, 0.18)** | |
| **Psychological Distress** |  |  |  |  | |
| Depression | 1.00 | 0.95 (0.82, 1.11) | 1.06 (0.91, 1.24) | 0.93 (0.78, 1.12) | |
| Depressive symptoms | 0.00 | -0.03 (-0.09, 0.02) | 0.01 (-0.06, 0.08) | -0.02 (-0.11, 0.06) | |
| Hopelessness | 0.00 | -0.05 (-0.10, -0.01)* | -0.08 (-0.14, -0.01)* | -0.10 (-0.17, -0.03)** | |
| Negative affect | 0.00 | -0.04 (-0.10, 0.02) | -0.04 (-0.11, 0.03) | -0.06 (-0.15, 0.03) | |
| Perceived constraints | 0.00 | -0.06 (-0.11, -0.02)** | -0.07 (-0.12, -0.01)* | -0.12 (-0.19, -0.06)** | |
| **Social Factors** |  |  |  |  | |
| Loneliness | 0.00 | -0.08 (-0.12, -0.03)** | -0.06 (-0.12, -0.00)* | -0.12 (-0.19, -0.06)** | |
| Not living with a spouse/partner | 1.00 | 1.03 (0.95, 1.11) | 1.07 (0.97, 1.18) | 1.10 (0.99, 1.21) | |
| Contact children ≥1x/week | 1.00 | 0.97 (0.91, 1.03) | 0.99 (0.92, 1.05) | 0.97 (0.90, 1.05) | |
| Contact other family ≥1x/week | 1.00 | 1.07 (0.98, 1.17) | 1.06 (0.96, 1.16) | 1.07 (0.96, 1.18) | |
| Contact friends ≥1x/week | 1.00 | 1.23 (1.14, 1.33)** | 1.30 (1.20, 1.40)** | 1.39 (1.27, 1.52)** | |

Abbreviations: CI, confidence interval; OR, odds ratio; RR, risk ratio.

^a^If the reference value is “1,” the effect estimate is OR or RR; if the reference value is “0,” the effect estimate is β.

^b^The analytic sample was restricted to those who had participated in the baseline wave (t_1_;2010 or 2012). Multiple imputation was performed to impute missing data on the exposure, covariates, and outcomes. All models controlled for sociodemographic characteristics (age, sex, race/ethnicity, marital status, annual household income, total wealth, level of education, employment status, health insurance, geographic region), pre-baseline childhood abuse, pre-baseline religious service attendance, pre-baseline values of the outcome variables (diabetes, hypertension, stroke, cancer, heart disease, lung disease, arthritis, overweight/obesity, physical functioning limitations, cognitive impairment, chronic pain, self-rated health, binge drinking, current smoking status, physical activity, sleep problems, positive affect, life satisfaction, optimism, purpose in life, mastery, health mastery, financial mastery, depressive symptoms, hopelessness, negative affect, perceived constraints, loneliness, living with spouse/partner, contact children <1x/week , contact other family <1x/week , contact friends <1x/week ), personality factors (openness, conscientiousness, extraversion, agreeableness, neuroticism) and the pre-baseline value of the exposure. These variables were controlled for in the pre-baseline was (in t0;2006 or 2008).

^c^We used an outcome-wide analytic approach and ran a separate model for each outcome. We ran a different type of model depending on the nature of the outcome: 1) for each binary outcome with a prevalence of ≥10%, we used a generalized linear model (with a log link and Poisson distribution) to estimate a RR; 2) for each binary outcome with a prevalence of <10%, we used a logistic regression model to estimate an OR; and 3) for each continuous outcome, we used a linear regression model to estimate a β.

^d^All continuous outcomes were standardized (mean=0; standard deviation=1), and β was the standardized effect size.

*p<0.05 before Bonferroni correction;**p<0.01 before Bonferroni correction;***p<0.05 after Bonferroni correction (the p-value cutoff for Bonferroni correction is p=0.05/35 outcomes=p<0.001).

Appendix Table 8. Friendship Network Quality (Negative Social Strain from Friends) and Subsequent Health and Well-being (Health and Retirement Study [HRS]: N=12,998)^a,b,c,d,e^

| **Outcomes** | **Friendship Network Quality (Negative Social Strain from Friends)** | | | |  |
| --- | --- | --- | --- | --- | --- |
|  | Quartile 1  (n=4,990) | Quartile 2  (n=2,368) | Quartile 3  (n=3,292) | Quartile 4  (n=2,338) | |
|  | (Reference) | RR/OR/β (95% CI) | RR/OR/β (95% CI) | RR/OR/β (95% CI) | |
| **Physical Health** |  |  |  |  | |
| All-cause mortality | 1.00 | 0.98 (0.84, 1.15) | 1.04 (0.90, 1.21) | 1.20 (1.00, 1.44)* | |
| Number of chronic conditions | 0.00 | 0.00 (-0.04, 0.04) | 0.01 (-0.02, 0.05) | 0.02 (-0.03, 0.08) | |
| Diabetes | 1.00 | 1.01 (0.91, 1.12) | 1.00 (0.91, 1.10) | 1.01 (0.89, 1.16) | |
| Hypertension | 1.00 | 1.00 (0.94, 1.07) | 1.01 (0.95, 1.07) | 1.00 (0.93, 1.07) | |
| Stroke | 1.00 | 1.01 (0.86, 1.18) | 1.00 (0.85, 1.19) | 1.09 (0.90, 1.31) | |
| Cancer | 1.00 | 0.98 (0.88, 1.10) | 0.99 (0.89, 1.10) | 0.96 (0.84, 1.10) | |
| Heart disease | 1.00 | 0.98 (0.89, 1.08) | 1.00 (0.92, 1.09) | 1.02 (0.92, 1.14) | |
| Lung disease | 1.00 | 1.11 (0.96, 1.29) | 1.09 (0.96, 1.25) | 1.16 (0.99, 1.36) | |
| Arthritis | 1.00 | 1.01 (0.95, 1.07) | 1.01 (0.96, 1.07) | 1.01 (0.94, 1.08) | |
| Overweight/obesity | 1.00 | 0.99 (0.93, 1.06) | 0.99 (0.93, 1.05) | 1.00 (0.93, 1.07) | |
| Physical functioning limitations | 1.00 | 1.01 (0.91, 1.12) | 1.00 (0.91, 1.10) | 1.04 (0.93, 1.15) | |
| Cognitive impairment | 1.00 | 0.94 (0.85, 1.05) | 0.93 (0.84, 1.02) | 1.01 (0.90, 1.13) | |
| Chronic pain | 1.00 | 1.03 (0.95, 1.11) | 0.97 (0.90, 1.05) | 0.99 (0.89, 1.09) | |
| Self-rated health | 0.00 | -0.01 (-0.06, 0.04) | -0.02 (-0.07, 0.03) | -0.03 (-0.09, 0.03) | |
| **Health Behaviors** |  |  |  |  | |
| Heavy drinking | 1.00 | 1.06 (0.79, 1.42) | 0.95 (0.72, 1.25) | 0.89 (0.67, 1.18) | |
| Smoking | 1.00 | 1.42 (1.02, 1.97)* | 0.97 (0.74, 1.26) | 0.95 (0.70, 1.30) | |
| Frequent physical activity | 1.00 | 1.01 (0.94, 1.08) | 0.99 (0.93, 1.05) | 0.99 (0.91, 1.06) | |
| Sleep problems | 1.00 | 1.01 (0.93, 1.09) | 1.01 (0.94, 1.09) | 1.00 (0.92, 1.10) | |
| **Psychological Well-Being** |  |  |  |  | |
| Positive affect | 0.00 | -0.03 (-0.08, 0.02) | -0.04 (-0.09, 0.01) | -0.08 (-0.13, -0.02)** | |
| Life satisfaction | 0.00 | -0.03 (-0.08, 0.02) | -0.03 (-0.08, 0.02) | -0.06 (-0.13, 0.02) | |
| Optimism | 0.00 | -0.01 (-0.06, 0.03) | -0.06 (-0.10, -0.02)** | -0.14 (-0.23, -0.05)** | |
| Purpose in life | 0.00 | -0.02 (-0.06, 0.02) | -0.01 (-0.05, 0.04) | -0.04 (-0.10, 0.01) | |
| Mastery | 0.00 | -0.04 (-0.09, 0.01) | -0.03 (-0.08, 0.02) | -0.08 (-0.15, -0.01)* | |
| Health mastery | 0.00 | -0.01 (-0.07, 0.05) | -0.02 (-0.08, 0.03) | -0.06 (-0.13, 0.00) | |
| Financial mastery | 0.00 | -0.01 (-0.07, 0.04) | -0.04 (-0.12, 0.03) | -0.10 (-0.22, 0.02) | |
| **Psychological Distress** |  |  |  |  | |
| Depression | 1.00 | 1.12 (0.96, 1.31) | 1.17 (1.00, 1.37) | 1.22 (1.02, 1.46)* | |
| Depressive symptoms | 0.00 | 0.04 (-0.00, 0.09) | 0.05 (-0.00, 0.10) | 0.11 (0.04, 0.17)** | |
| Hopelessness | 0.00 | 0.01 (-0.05, 0.07) | 0.03 (-0.01, 0.07) | 0.10 (0.04, 0.17)** | |
| Negative affect | 0.00 | 0.07 (0.03, 0.11)** | 0.14 (0.10, 0.18)** | 0.28 (0.21, 0.35)** | |
| Perceived constraints | 0.00 | 0.04 (-0.02, 0.10) | 0.05 (0.01, 0.10)* | 0.12 (0.01, 0.23)* | |
| **Social Factors** |  |  |  |  | |
| Loneliness | 0.00 | 0.07 (0.03, 0.12)** | 0.11 (0.06, 0.16)** | 0.19 (0.09, 0.30)** | |
| Not living with a spouse/partner | 1.00 | 1.02 (0.94, 1.11) | 0.97 (0.89, 1.05) | 0.96 (0.87, 1.05) | |
| Contact children ≥1x/week | 1.00 | 1.01 (0.94, 1.07) | 1.00 (0.94, 1.06) | 0.99 (0.93, 1.06) | |
| Contact other family ≥1x/week | 1.00 | 0.98 (0.92, 1.06) | 1.01 (0.95, 1.08) | 1.02 (0.92, 1.12) | |
| Contact friends ≥1x/week | 1.00 | 1.02 (0.96, 1.09) | 1.02 (0.96, 1.09) | 1.04 (0.96, 1.13) | |

Abbreviations: CI, confidence interval; OR, odds ratio; RR, risk ratio.

^a^If the reference value is “1,” the effect estimate is OR or RR; if the reference value is “0,” the effect estimate is β.

^b^The analytic sample was restricted to those who had participated in the baseline wave (t_1_;2010 or 2012). Multiple imputation was performed to impute missing data on the exposure, covariates, and outcomes. All models controlled for sociodemographic characteristics (age, sex, race/ethnicity, marital status, annual household income, total wealth, level of education, employment status, health insurance, geographic region), pre-baseline childhood abuse, pre-baseline religious service attendance, pre-baseline values of the outcome variables (diabetes, hypertension, stroke, cancer, heart disease, lung disease, arthritis, overweight/obesity, physical functioning limitations, cognitive impairment, chronic pain, self-rated health, binge drinking, current smoking status, physical activity, sleep problems, positive affect, life satisfaction, optimism, purpose in life, mastery, health mastery, financial mastery, depressive symptoms, hopelessness, negative affect, perceived constraints, loneliness, living with spouse/partner, contact children <1x/week , contact other family <1x/week , contact friends <1x/week ), personality factors (openness, conscientiousness, extraversion, agreeableness, neuroticism) and the pre-baseline value of the exposure. These variables were controlled for in the pre-baseline was (in t0;2006 or 2008).

^c^We used an outcome-wide analytic approach and ran a separate model for each outcome. We ran a different type of model depending on the nature of the outcome: 1) for each binary outcome with a prevalence of ≥10%, we used a generalized linear model (with a log link and Poisson distribution) to estimate a RR; 2) for each binary outcome with a prevalence of <10%, we used a logistic regression model to estimate an OR; and 3) for each continuous outcome, we used a linear regression model to estimate a β.

^d^All continuous outcomes were standardized (mean=0; standard deviation=1), and β was the standardized effect size.

^e^In these analyses, negative social strain from friends was not reverse coded. Thus, higher scores indicated more negative social strain from friends.

*p<0.05 before Bonferroni correction;**p<0.01 before Bonferroni correction;***p<0.05 after Bonferroni correction (the p-value cutoff for Bonferroni correction is p=0.05/35 outcomes=p<0.001).
